# Progression and Regression of Chronic Kidney Disease by Age Among Adults in a Population-Based Cohort in Alberta, Canada

**DOI:** 10.1001/jamanetworkopen.2021.12828

**Published:** 2021-06-08

**Authors:** Ping Liu, Rob R. Quinn, Ngan N. Lam, Huda Al-Wahsh, Manish M. Sood, Navdeep Tangri, Marcello Tonelli, Pietro Ravani

**Affiliations:** 1 Department of Medicine, Cumming School of Medicine, University of Calgary, Calgary, Alberta, Canada; 2 Department of Medicine, Ottawa Hospital Research Institute, The Ottawa Hospital, University of Ottawa, Ottawa, Ontario, Canada; 3 Department of Medicine, Seven Oaks General Hospital, University of Manitoba, Winnipeg, Canada; 4 Department of Community Health Sciences, Seven Oaks General Hospital, University of Manitoba, Winnipeg, Canada

## Abstract

**Importance:**

The burden of chronic kidney disease (CKD) is expected to increase worldwide as the global population ages, potentially increasing the demand for nephrology services. Understanding whether CKD inevitably progresses or may regress can inform clinical decision-making and health policy.

**Objective:**

To study CKD progression and regression by age in adults with CKD.

**Design, Setting, and Participants:**

This population-based cohort study used linked administrative and laboratory data to assess adults in the province of Alberta, Canada, with incident mild, moderate, or severe CKD, defined by estimated glomerular filtration rate (eGFR) of 45 to 59, 30 to 44, or 15 to 29 mL/min/1.73 m^2^ for longer than 3 months, from April 1, 2009, to March 31, 2015. Data were analyzed from July 20 to November 30, 2020.

**Exposures:**

Age.

**Main Outcomes and Measures:**

Time to the earliest of CKD regression or progression (defined as sustained increase or drop in eGFR category for >3 months, respectively, and a ≥25% increase or decrease in eGFR from baseline, respectively), kidney failure (the earlier of kidney replacement initiation or eGFR <15 mL/min/1.73 m^2^ for >3 months), death, or censoring (outmigration, 5 years of follow-up, or end of study on March 31, 2017).

**Results:**

Study participants with CKD (55.2% women and 44.8% men) included 81 320 with mild CKD (mean [SD] age, 72.4 [11.3] years), 35 929 with moderate CKD (mean [SD] age, 77.1 [11.5] years), and 12 237 with severe CKD (mean [SD] age, 76.6 [13.8] years). The annual incidence of CKD increased with advancing age, from 180 per 100 000 population younger than 65 years to 7250 per 100 000 in those 85 years or older. After cohort entry, the 5-year probability of regression was similar to that of progression or kidney failure in mild (14.3% vs 14.6%), moderate (18.9% vs 16.5%), and severe (19.3% vs 20.4%) CKD. As mortality at 5 years increased with advancing age in moderate (from 9.6% for age <65 years to 48.4% for age ≥85 years) and severe (from 10.8% for age <65 years to 60.2% for age ≥85 years) CKD, the risk of progression or kidney failure decreased substantially (for moderate CKD, from 32.3% for <65 years to 9.4% for ≥85 years; for severe CKD, from 55.2% for <65 years to 4.7% for ≥85 years), whereas the probabilities of regression varied to a lesser extent (for moderate CKD, from 22.5% for <65 years to 15.4% for ≥85 years; for severe CKD, from 13.9% for <65 years to 18.7% for ≥85 years).

**Conclusions and Relevance:**

This cohort study found that with advancing age, CKD regression and death were more likely than CKD progression or kidney failure. These findings have important implications for patient care and for assessing the potential effect of population aging on the burden of CKD.

## Key Points

**Question:**

How likely is regression of mild to severe chronic kidney disease (CKD) in adults?

**Findings:**

In this population-based cohort study of adults with incident CKD, the 5-year probability of regression was similar to that of progression or kidney failure in mild (14.3% vs 14.6%), moderate (18.9% vs 16.5%), and severe (19.3% vs 20.4%) CKD. As the risk of death increased with advancing age, the risk of progression decreased substantially, whereas the probabilities of regression varied to a lesser extent and exceeded the risk of progression in moderate or severe CKD.

**Meaning:**

These findings suggest that CKD regression should be considered in the allocation of health resources and in patient counseling.

## Teaser

This population-based cohort study assesses progression and regression of disease among adults with chronic kidney disease in Alberta, Canada.

## Introduction

The number of people 65 years and older is projected to more than double from 703 million in 2019 to more than 1.5 billion in 2050.^1^ With population aging, the burden of most age-related conditions, including chronic kidney disease (CKD), is expected to increase. Chronic kidney disease is a worldwide public health challenge associated with significant morbidity, mortality, and high health care costs.^2,3^ The expected increasing incidence and prevalence of CKD with global population aging may put additional pressures on current limited capacity for the delivery of kidney health care worldwide.^4,5,6^

Chronic kidney disease is often considered a condition that tends to progress over time.^7^ Although referral to a nephrologist is recommended for progressive CKD or for estimated glomerular filtration rate (eGFR) of less than 30 mL/min/1.73 m^2^, patients with less severe CKD are increasingly referred for specialist nephrology care,^7,8,9,10^ perhaps in anticipation that such care will eventually be required. When eGFR improves to greater than 30 mL/min/1.73 m^2^, discharge from nephrology clinics back to primary care occurs infrequently,^11^ partly because of the limited data that are available on the frequency and outcomes of such improvements,^12,13,14^ which are occasionally referred to as *CKD regression*.^15^ Understanding how often CKD regression occurs and its outcomes and whether CKD regression varies by age may inform clinical decision-making and health policy, including referral recommendations.

We studied the outcomes of CKD regression and progression in an incident cohort of adults with mild to severe CKD using population-based data. We quantified the probabilities of sustained kidney function improvement (regression), progression, kidney failure, and death, and how these outcomes varied with age. We also assessed the outcomes of participants who experienced CKD regression. Analyses were designed to minimize regression of CKD after acute kidney injury or owing to biological variability in eGFR measurements.

## Methods

### Study Design and Data Sources

We conducted a population-based cohort study using linked administrative and laboratory data from Alberta, Canada. The Alberta Health database contains information on demographics, vital statistics, diagnostics, and procedures for inpatient and outpatient health services.^16^ More than 99% of Alberta residents are registered with Alberta Health and have universal access to hospital care, laboratory testing, and physician services (including dialysis and kidney transplant). The institutional review boards at the Universities of Alberta and Calgary approved the study with a waiver of participant consent because retrospective deidentified data were used. We followed the reporting standards recommended by Reporting of Studies Conducted Using Observational Routinely-Collected Health Data (RECORD)^17^ and used definitions from Kidney Disease: Improving Global Outcomes^18^ when reporting kidney function and CKD. Data were analyzed from July 20 to November 30, 2020.

We calculated eGFR using the Chronic Kidney Disease Epidemiology (CKD-EPI) equation, with serum creatinine values standardized to the isotope-dilution mass spectrometry reference method.^19^ We assumed that all participants were White, because data on race were not available and misclassification of eGFR is expected to be minimal in Alberta, where only 3% of the population is Black.^20^ We used only outpatient serum creatinine measurements to enhance the generalizability of findings to general practices and minimize the inclusion of people with episodes of acute kidney injury. We used the mean value of eGFR when there were multiple measurements on the same day.

### CKD Cohorts

We included individuals 18 years or older with incident mild (eGFR, 45-59 mL/min/1.73 m^2^ [stage G3a]), moderate (eGFR, 30-44 mL/min/1.73 m^2^ [stage G3b]), and severe (eGFR, 15-29 mL/min/1.73 m^2^ [stage G4]) CKD. We followed guideline recommendations to define CKD based on confirmed eGFR reduction for longer than 3 months.^7^ To determine each individual’s eligibility to enter a CKD stage and reduce the effect of regression to the mean,^21^ we screened all consecutive series of 2 or more eGFR measurements, where the first and the last eGFR were separated by more than 90 days and all intervening measurements were within 90 days. We implemented a sustained eGFR method to select the earliest series (qualifying period) for cohort entry, whereby the last eGFR (index eGFR) in the qualifying period defined the corresponding CKD stage and all eGFR values in that series of measurements were below the upper limit of the defined CKD stage (eFigure 1 in the Supplement). The date of the last eGFR in a qualifying period defined the index date (cohort entry), from April 1, 2009, to March 31, 2015. We used data on eGFR (from May 1, 2002) and kidney replacement therapies (from April 1, 1994) to exclude individuals who had previously qualified for a more severe stage or initiated kidney replacement therapy (eMethods and eTables 1 and 2 in the Supplement).

### Outcomes and Independent Variables

The primary outcome was time to the earliest of sustained kidney function improvement (regression), CKD progression (progression), kidney failure, or death, which we treated as competing events. We defined kidney outcomes using the same methods we implemented to define stage entry (eTable 1 in the Supplement). We defined regression as a sustained, higher eGFR category for longer than 3 months and a 25% or greater increase in the eGFR from baseline. We defined progression as a sustained drop in eGFR category for longer than 3 months and a 25% or greater decrease in the eGFR from baseline.^7^ The date of the last eGFR during a qualifying period defined the date of regression or progression. The date of kidney failure was the earlier date of initiation of long-term kidney replacement therapy (determined from provincial registration) or eGFR of less than 15 mL/min/1.73 m^2^ for longer than 3 months. When these 2 events occurred on the same day, we defined the outcome as kidney replacement therapy. We ascertained death from Alberta Vital Statistics.^16^ For the subset of individuals who experienced CKD regression, we studied the competing outcomes of further regression, progression, kidney failure, and death (eTable 1 in the Supplement). We censored observations at the date of emigration from the province, study end (March 31, 2017), or after 5 years of observation. We described 3 CKD cohorts considering baseline age (exposure), sex, index eGFR, duration of the qualifying period, number of eGFR measurements during the qualifying period, prior outpatient eGFR measurements, albuminuria, comorbidities, indicators of acute conditions, or receipt of potentially nephrotoxic procedures within 3 months before cohort entry (hospitalizations, emergency department visits, or receipt of angiogram or cardiac catheterization), and drugs dispensed within the year before cohort entry (eMethods and eTable 3 in the Supplement).

### Statistical Analysis

We performed identical and separate analyses for each cohort. We estimated the incidence of CKD using population data from Statistics Canada.^22^ We summarized data recorded during the qualifying period and baseline characteristics using standard descriptive statistics. We used the nonparametric Aalen-Johansen method^23^ to estimate the cumulative incidence functions of competing events and reported visual summaries of these functions across categories defined by CKD stage and age. We used the same approach to study the outcomes in those who experienced CKD regression.

We performed analyses stratified by other key characteristics (severity of albuminuria, dispensation of renal protective drugs, and comorbidities) and in restricted samples (stable CKD for 1 or 2 years, absence of prior acute conditions, or receipt of potentially nephrotoxic procedures) (eMethods in the Supplement). We used the Kaplan-Meier method to assess how inclusion (vs exclusion) of regression among the reasons to leave a CKD stage may affect the estimate of time spent in that stage (disease duration).

### Sensitivity Analyses

We repeated all analyses by using a chronicity criterion of 3 to 15 months for cohort creation and outcome definition, as opposed to longer than 3 months without upper limit. We repeated all analyses using the moving average eGFR method instead of the sustained eGFR method (eMethods in the Supplement).

## Results

### Study Cohorts

Study participants with mild CKD represented the largest cohort (n = 81 320), followed by those with moderate (n = 35 929) and severe (n = 12 237) CKD (cohort formation details are provided in eTable 4 and eFigure 2 in the Supplement). More severe CKD stages included individuals who were older (mean [SD] age for mild CKD, 72.4 [11.3] years; for moderate CKD, 77.1 [11.5] years; and for severe CKD, 76.6 [13.8] years), had shorter qualifying periods for cohort entry (median for mild CKD, 255 [interquartile range (IQR), 144-415] days; for moderate CKD, 179 [IQR, 117-324] days; and for severe CKD, 133 [IQR, 104-208] days), and had more comorbidities (eg, diabetes was present in 29.1% with mild CKD, 40.0% with moderate CKD, and 51.5% with severe CKD; cardiovascular disease was present in 31.1% with mild CKD, 47.6% with moderate CKD, and 57.7% with severe CKD). Severe albuminuria was more common in more severe CKD (27.7% vs 4.9% for mild CKD and 10.8% for moderate CKD) and less common with advancing age (severe albuminuria in those aged <65 years vs ≥85 years, 8.7% vs 3.4% for mild CKD, 28.4% vs 5.1% for moderate CKD, and 58.4% vs 12.0% for severe CKD) (Table and eFigure 3 in the Supplement). Follow-up information is summarized in eTable 5 in the Supplement. Participants who were older or had less severe CKD tended to have lower rates of outpatient eGFR measurements during follow-up (incidence rate ratio among those aged ≥85 years, 0.88 [95% CI, 0.86-0.90] for mild CKD vs 0.64 [95% CI, 0.62-0.66] for moderate CKD and 0.64 [95% CI, 0.61-0.67] for severe CKD) (eTable 6 in the Supplement). Follow-up data on eGFR measurements are reported in eTable 7 in the Supplement.

**Table.** Baseline Characteristics by CKD Stage

Characteristic	CKD Stage^a^		
	Mild (n = 81 320)	Moderate (n = 35 929)	Severe (n = 12 237)
Age, mean (SD), y	72.4 (11.3)	77.1 (11.5)	76.6 (13.8)
Age group, y			
18-64	19 674 (24.2)	4931 (13.7)	2196 (17.9)
65-74	26 071 (32.1)	8238 (22.9)	2268 (18.5)
75-84	25 377 (31.2)	13 531 (37.7)	4134 (33.8)
≥85	10 198 (12.5)	9229 (25.7)	3639 (29.7)
Sex			
Women	44 861 (55.2)	20 105 (56.0)	6543 (53.5)
Men	36 459 (44.8)	15 824 (44.0)	5694 (46.5)
Kidney health measures			
Index eGFR, mean (SD), mL/min/1.73 m^2^	53.9 (4.1)	39.0 (4.0)	24.6 (3.8)
Qualifying period, median (IQR), d	255 (144-415)	179 (117-324)	133 (104-208)
Qualifying period, d			
≤365	54 444 (67.0)	28 771 (80.1)	11 101 (90.7)
366-455	14 242 (17.5)	3978 (11.1)	657 (5.4)
456-730	5168 (6.4)	1443 (4.0)	233 (1.9)
>730	7466 (9.2)	1737 (4.8)	246 (2.0)
Qualifying period, No. of outpatient eGFR measurements, mean (SD)	2 (1)	3 (2)	3 (2)
Prior outpatient eGFR, mL/min/1.73 m^2^			
Unmeasured	6387 (7.9)	1722 (4.8)	396 (3.2)
≥60	70 415 (86.6)	3583 (10.0)	253 (2.1)
45 to <60	4158 (5.1)	28 971 (80.6)	752 (6.1)
30 to <45	334 (0.4)	1537 (4.3)	10 677 (87.3)
15 to <30	26 (0.03)	116 (0.3)	159 (1.3)
Albuminuria^b^			
Unmeasured	4169 (5.1)	1588 (4.4)	377 (3.1)
Normal or mild	63 141 (77.6)	23 361 (65.0)	5254 (42.9)
Moderate	10 041 (12.3)	7117 (19.8)	3217 (26.3)
Severe	3969 (4.9)	3863 (10.8)	3389 (27.7)
Comorbidities			
Diabetes	23 649 (29.1)	14 383 (40.0)	6298 (51.5)
Hypertension	62 605 (77.0)	32 338 (90.0)	11 457 (93.6)
Cardiovascular disease	25 279 (31.1)	17 090 (47.6)	7064 (57.7)
Congestive heart failure	11 977 (14.7)	10 104 (28.1)	4931 (40.3)
Myocardial infarction	5208 (6.4)	3553 (9.9)	1492 (12.2)
Peripheral vascular disease	2949 (3.6)	2298 (6.4)	1010 (8.3)
Stroke or TIA	12 775 (15.7)	8186 (22.8)	3265 (26.7)
Cancer	11 148 (13.7)	5837 (16.2)	2070 (16.9)
Lymphoma	1092 (1.3)	664 (1.8)	280 (2.3)
Metastatic	2586 (3.2)	1415 (3.9)	516 (4.2)
Nonmetastatic	9562 (11.8)	4908 (13.7)	1700 (13.9)
Dementia	5812 (7.1)	4310 (12.0)	1758 (14.4)
Indicators of acute conditions			
Hospitalization	5034 (6.2)	3539 (9.8)	1830 (15.0)
Emergency department visit	10 963 (13.5)	6673 (18.6)	2931 (24.0)
Receipt of angiogram or cardiac catheterization	573 (0.7)	332 (0.9)	87 (0.7)
Drugs dispensed			
ACEI or ARB	49 598 (61.0)	27 362 (76.2)	9716 (79.4)
Statins	34 418 (42.3)	17 936 (49.9)	6771 (55.3)
NSAIDs	17 948 (22.1)	7649 (21.3)	1753 (14.3)

Abbreviations: ACEI, angiotensin-converting enzyme inhibitor; ARB, angiotensin receptor blocker; CKD, chronic kidney disease; eGFR, estimated glomerular filtration rate; IQR, interquartile range; NSAID, nonsteroidal anti-inflammatory drug; TIA, transient ischemic attack.

^a^ Unless otherwise indicated, data are expressed as No. (%) of patients. Percentages have been rounded and may not total 100.

^b^ Categorized as normal/mild, moderate, severe, or unmeasured, based on the most recent outpatient values, with the following types of measurement in descending order of preference: albumin-to-creatinine ratio (<30, 30-300, or >300 mg/g), protein-to-creatinine ratio (<150, 150-500, or >500 mg/g), and urine dipstick (negative or trace finding, 1+, or ≥2+).

### Incidence Rate

**Figure 1.**
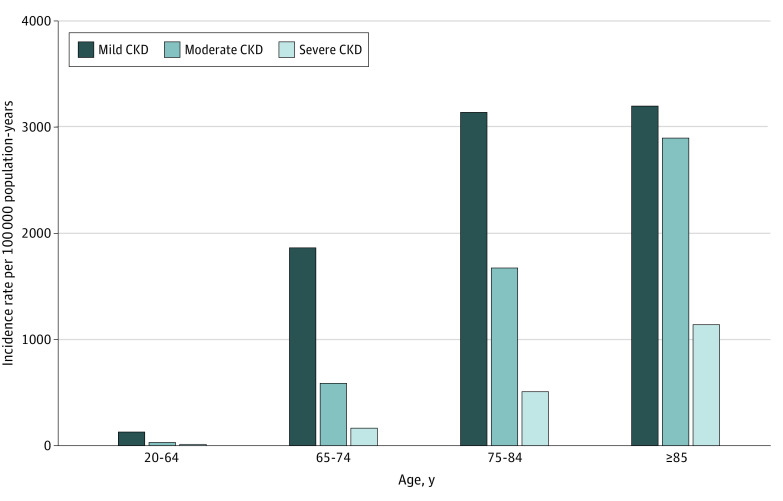
Incidence of Chronic Kidney Disease (CKD) by Stage and Patient Age We measured the incidence of CKD using new cases of CKD (by stage) among adults in Alberta who had estimated glomerular filtration rate measurements (numerator) and estimates of the Alberta population (by age and year) from Statistics Canada (denominator).

The incidence of mild to severe CKD during the study period increased with older age, from 180 per 100 000 population younger than 65 years to 7250 per 100 000 in those 85 years or older. The incidence rates of mild CKD were 132 per 100 000 population-years in individuals aged 20 to 64 years, 1870 per 100 000 population-years in individuals aged 65 to 74 years, 3144 per 100 000 population-years in individuals aged 75 to 84 years, and 3206 per 100 000 population-years in individuals who were 85 years or older. Incidence rates of moderate CKD (33 per 100 000 population-years in individuals aged 20-64 years, 591 per 100 000 population-years in individuals aged 65-74 years, 1676 per 100 000 population-years in individuals aged 75-84 years, and 2901 per 100 000 population-years in individuals aged ≥85 years) and severe CKD (15 per 100 000 population-years in individuals aged 20-64 years, 163 per 100 000 population-years in individuals aged 65-74 years, 512 per 100 000 population-years in individuals aged 75-84 years, and 1144 per 100 000 population-years in individuals aged ≥85 years) were lower than those for mild CKD but increased more steeply with advancing age (Figure 1).

### Outcomes of Initial CKD Stage

#### Outcomes by CKD Stage

**Figure 2.**
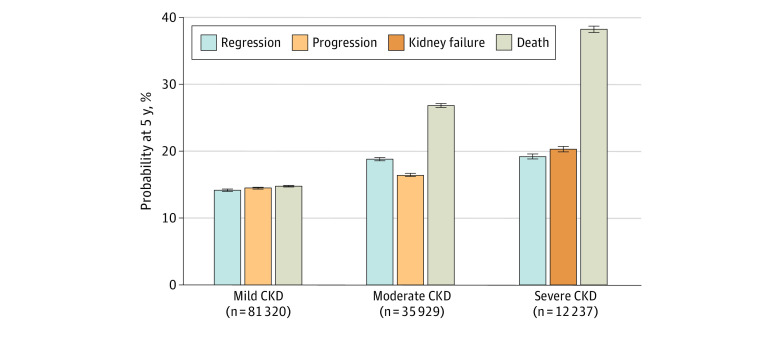
Outcome Probabilities at 5 Years From Study Entry Outcome probabilities were estimated using cumulative incidence functions at 5 years after study entry. Progression represents chronic kidney disease (CKD) progression or kidney failure. The 5-year probabilities of CKD progression and kidney failure were 14.5% and less than 0.01%, respectively, for individuals with mild CKD and 16.2% and 0.3%, respectively, for individuals with moderate CKD.

Within each stage, the 5-year probabilities of regression were comparable to the risk of progression or kidney failure (Figure 2 and eFigure 4 in the Supplement). With worse CKD stage, 5-year mortality increased to a larger extent (14.9% for mild CKD, 26.9% for moderate CKD, and 38.2% for severe CKD) than progression or kidney failure (14.6% for mild CKD, 16.5% for moderate CKD, and 20.4% for severe CKD) and regression (14.3% for mild CKD, 18.9% for moderate CKD, and 19.3% for severe CKD).

#### Analyses Stratified by Age

**Figure 3.**
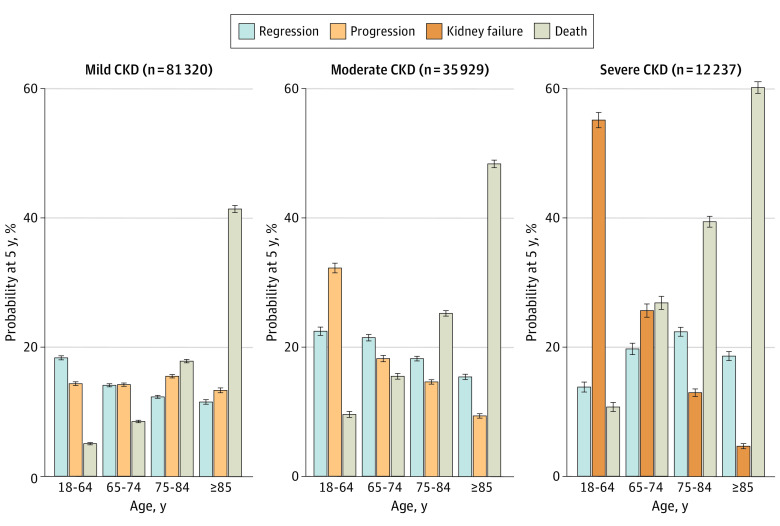
Outcome Probabilities at 5 Years From Study Entry by Age Outcome probabilities were estimated using cumulative incidence functions at 5 years after study entry, stratified by categories of baseline age. Progression represents chronic kidney disease (CKD) progression or kidney failure. The 5-year probabilities of kidney failure were less than 0.1% for individuals with mild CKD, regardless of age; among individuals with moderate CKD, probabilities were 1.3% for those aged 18 to 64 years, 0.3% for those aged 65 to 74 years, 0.1% for those aged 75 to 84 years, and 0.1% for those 85 years or older.

Regression was observed in all groups defined by age and CKD severity, with 5-year probabilities ranging from 12% to 22%. With advancing age, the probability of regression decreased to a lesser extent (for moderate CKD, from 22.5% for <65 years to 15.4% for ≥85 years; for severe CKD, from 13.9% for <65 years to 18.7% for ≥85 years) than the risk of progression or kidney failure (for moderate CKD, from 32.3% for <65 years to 9.4% for ≥85 years; for severe CKD, from 55.2% for <65 years to 4.7% for ≥85 years) in moderate to severe CKD (Figure 3). With more advanced stages of CKD, the increased risk of death with advancing age was more pronounced (for moderate CKD, from 9.6% for <65 years to 48.4% for ≥85 years; for severe CKD, from 10.8% for <65 years to 60.2% for ≥85 years).

#### Subgroup Analyses

The probability of regression was lower in individuals with severe albuminuria, regardless of age and CKD stage, although the pattern of association between outcomes and age was similar. For example, the probability of regression at 5 years for moderate CKD was 32.3% for mild or normal albuminuria vs 7.7% for severe albuminuria in individuals younger than 65 years and 17.2% for mild or normal albuminuria vs 9.2% for severe albuminuria in individuals 85 years or older (eFigure 5 in the Supplement). Results were similar in analyses restricted to individuals without prior acute conditions or receipt of potentially nephrotoxic procedures (eFigures 6-9 in the Supplement) and participants who were still in the initial CKD stage at 1 and 2 years (eFigure 10 in the Supplement). The probabilities of regression by age were similar according to dispensation of renal protective drugs (eFigure 11 in the Supplement) or comorbidity groups defined by diabetes and cardiovascular disease (eFigure 12 in the Supplement).

### Outcomes After CKD Regression

A total of 6775 individuals who experienced CKD regression from mild CKD (67.4%) still had sustained eGFR of greater than 60 mL/min/1.73 m^2^ at 5 years and had similar risks of progression (28.5%) and death (25.0%). Further regression occurred in some individuals with regression from moderate (5-year probability, 7.1%) or severe CKD (5-year probability, 6.5%), although most remained in the improved stage, experienced progression, or died (eFigures 13 and 14 in the Supplement). Individuals who experienced regression were more likely to remain in the improved stage or experience further improvement than progression, regardless of age and CKD severity (eFigure 15 in the Supplement). The median number of outpatient eGFR measurements in those who remained event-free for 5 years after regression from mild CKD was 24 (IQR, 17-37), from moderate CKD, 30 (IQR, 21-44); and from severe CKD, 41 (IQR, 27-63).

### Disease Duration

**Figure 4.**
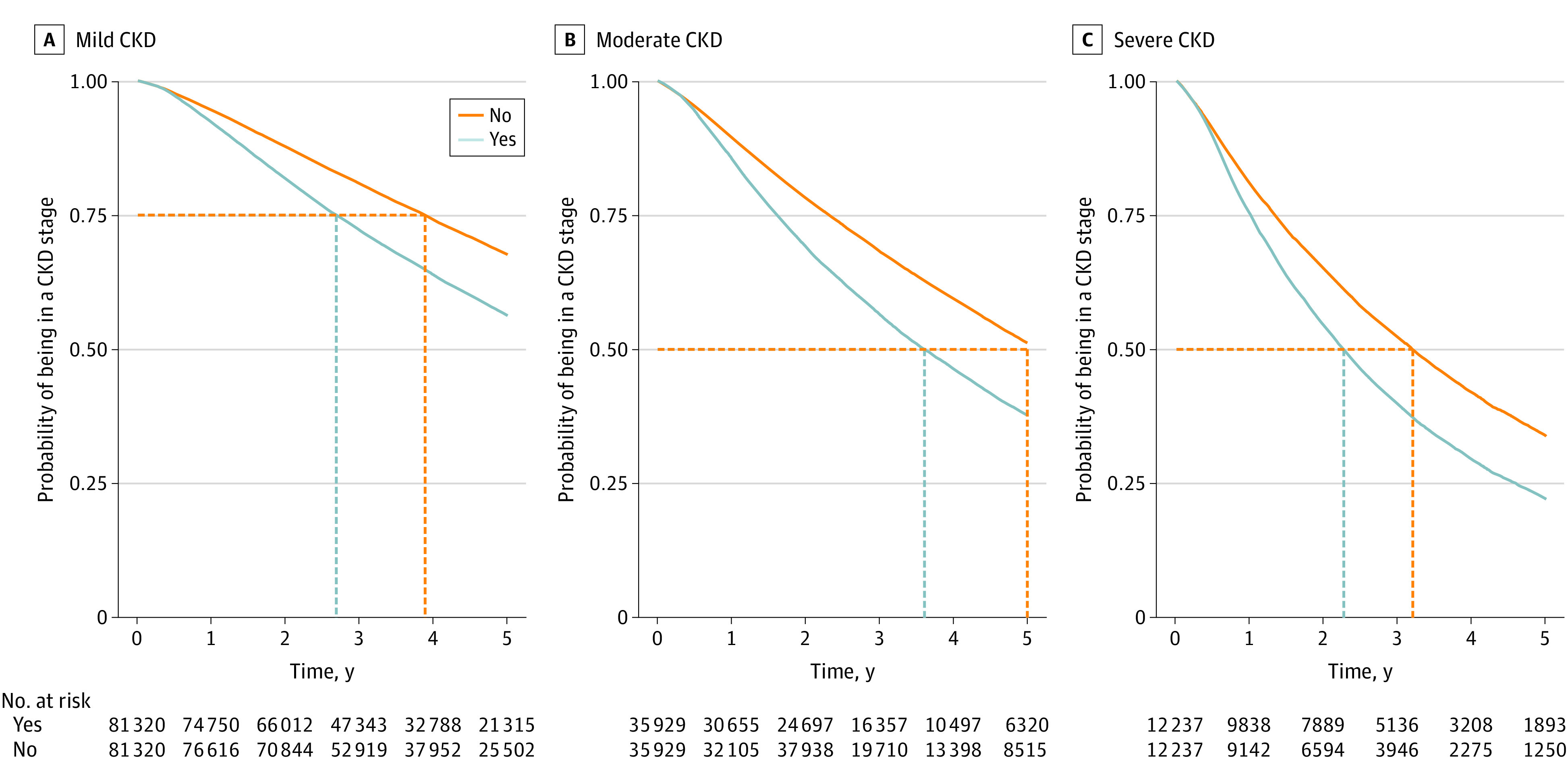
Time Spent in a Chronic Kidney Disease (CKD) Stage Time spent in a CKD stage (disease duration) was estimated using the Kaplan-Meier method, comparing inclusion of CKD regression (Yes) vs exclusion of CKD regression (No) among the reasons to leave a CKD stage. The vertical dotted lines indicate time spent in a corresponding CKD stage in 75% of the individuals with mild CKD (A), in 50% of the individuals with moderate CKD (B), and in 50% of the individuals with severe CKD (C).

Time spent in the initial CKD stage was longer when regression was not considered among the reasons to leave that stage. Without considering regression as a reason to leave a CKD stage, the median time spent in severe CKD increased from 2.3 (IQR, 1.0-4.6) to 3.2 (IQR, 1.3 to >5.0) years and the median time spent in moderate CKD increased from 3.6 (IQR, 1.6 to >5.0) to longer than 5.0 years (Figure 4).

### Sensitivity Analyses

We found similar results in analyses based on the moving average eGFR method or applying an upper limit of 15 months to the chronicity criterion. Details are provided in eTable 8 and eFigures 16 to 20 in the Supplement.

## Discussion

In this population-based cohort study of adults with incident CKD, regression was observed in all groups defined by age and CKD severity, with 5-year probabilities ranging from 12% to 22%. Three important findings emerged. First, regression was at least as common as progression or kidney failure in mild to severe CKD. Second, as the competing risk of death increased with advancing age, the probability of regression decreased to a lesser extent than the risk of progression or kidney failure in moderate to severe CKD. Finally, after regression, stable CKD or further regression was more likely than progression, regardless of age or CKD severity.

Chronic kidney disease is often viewed as a chronic and irreversible condition with a lifelong course.^7^ Stability of kidney function over time is seen as the best-case scenario for a person with CKD. In contrast to this perception, we found that the probability of CKD regression was similar or higher than the risk of progression or kidney failure among adults 65 years or older with mild to severe CKD. Chronic kidney disease regression may be due to regression to the mean, recovery from episodes of acute kidney injury, GFR-independent factors (loss of muscle mass, cachexia, or dietary changes over time),^24^ or true improvement because of risk factor modification by treatment or spontaneous recovery. By using 2 or more eGFR measurements longer than 90 days apart when forming CKD cohorts and identifying outcomes, we attempted to estimate each individual’s true mean kidney function over time by minimizing the effect of regression to the mean.^21^ Although some participants could have recovered from acute kidney injury rather than experienced regression of CKD, the former is less likely in our study, because we applied a chronicity criterion of longer than 3 months to identify CKD cohorts and kidney outcomes and used only outpatient eGFR measurements. Our findings were confirmed in subgroup analyses that excluded participants at risk for acute kidney injury (previous hospitalization or receipt of potentially nephrotoxic procedures), illness, or amputation with possible muscle mass reduction (previous hospitalization)^25^ or included only those with stable CKD for at least 1 year. Similar probabilities of sustained kidney function improvement across CKD stages, including mild CKD, are consistent with findings from animal models and in patients with diabetes after pancreatic transplant.^15^

Because the competing risk of death increased with advancing age, the risk of progression or kidney failure decreased to a larger extent than the probability of regression. A potential explanation is that in the absence of severe albuminuria, CKD in older adults may result from multiple chronic disease–causing mechanisms leading to accelerated frailty before progression or kidney failure can ensue,^26^ whereas CKD regression may still occur in older adults who are less frail. Our finding of reduced probability of regression in people with severe albuminuria supports this hypothesis.

This study further expands our previous work on the association between age and kidney failure and death in adults with severe CKD.^4^ With advancing age, the risk of progression or kidney failure decreased in moderate to severe CKD, whereas regression was at least as likely as progression or kidney failure in mild to severe CKD stages. Our findings are consistent with a study of mild to moderate CKD in primary care (n = 1741) (mean eGFR, 53.5 mL/min/1.73 m^2^)^12^ in which most people (34%) had stable kidney function, only a very small fraction (0.2%) developed kidney failure, 18% had progression, and 19% had no evidence of CKD after 5 years. Other studies of patients with hypertensive CKD or receiving nephrology care^13,14^ also reported CKD regression. To our knowledge, our study is the first to assess the incidence and outcomes of incident mild, moderate, and severe CKD across categories of age, as well as the outcomes after CKD regression using population-based data.

This study has clinical practice, health policy, and research implications. First, CKD regression is an important outcome to discuss along with progression, kidney failure, and death in clinical decision-making and advance care planning. Considerations of both adverse and favorable outcomes will provide patients with a more comprehensive view of the disease trajectory and promote optimism and hope, with potentially positive psychological effects that may enhance patient engagement in CKD management.^27^ Second, for adults who are younger than 65 years or have severe albuminuria, the high risk of progression or kidney failure and the low probability of regression warrant more intensive monitoring and treatment, even in less severe stages. Considering the extremely low incidence of mild to moderate CKD in adults younger than 65 years (165 per 100 000 population per year) and the observation that only 5% to 11% of them have severe albuminuria, nephrology consultation in this patient group is not expected to overwhelm nephrology clinics. Most of the remaining CKD population, which includes older adults (aged ≥65 years) with less severe albuminuria and thus higher probability of regression than progression, includes many candidates for discharge from nephrology clinics to primary care. Finally, our study highlights the importance of considering the outcome of regression, regardless of age and CKD severity, and has implications for research using readily available, community-based laboratory data to study CKD burden and prognosis. Ignoring the possibility of regression will result in overestimation of disease duration and thus disease prevalence in modeling studies.

This study has several strengths, including its community-based setting in a geographically defined population with universal access to health care (including serum creatinine assessment and kidney replacement). Furthermore, we applied a chronicity criterion of longer than 3 months to minimize regression to the mean when forming CKD cohorts and identifying outcomes.^7^ We also used a long washout period to maximize the inclusion of incident cases. Finally, the large sample size and extended follow-up allowed us to explore how the probabilities of favorable and adverse outcomes varied by age and across CKD stages. Although regression to the mean does happen and progression takes time to occur, a 5-year observation period should have been long enough to capture these outcomes at the population level.

### Limitations

Our study has some limitations. First, we used routinely collected laboratory data, and thus many factors could have influenced the frequency of eGFR measurements, including the severity of CKD, age, and prognosis. Generally, older individuals or those at low risk of progression are assessed less often. If kidney outcomes were classified using prespecified prospective data, we would arguably have detected a higher probability of CKD regression, especially in older individuals or in those with less severe CKD, and thus this limitation is unlikely to have threatened our conclusions. Second, the observed CKD regression may be explained by changes in muscle mass or diet. However, we obtained similar results after excluding people with previous hospitalization, including those who may have lost muscle mass because of illness or limb amputation. After CKD regression, mortality was less likely than stable CKD or further regression. Third, the study source population was predominantly White, and this may limit generalization of our findings to populations with different ethnic composition. The study population was based on the CKD-EPI equation and included stages G3a, G3b, and G4 CKD; thus our findings may not apply when other methods are used to estimate GFR or to earlier stages of CKD.

## Conclusions

According to this population-based cohort study, CKD regression may be at least as common as CKD progression or kidney failure for mild to severe CKD. Although the incidence of CKD increases with advancing age, the probability of CKD regression and mortality far exceeded the risk of progression or kidney failure. Therefore, the aging of the general population may not necessarily translate into increased CKD burden for patients and health services.

## Notes

Supplement. **eMethods.** CKD Cohorts, Average eGFR Method, and Covariates **eTable 1.** Methods for Cohort Formation and Outcome Definition **eTable 2.** Codes for Identifying Dialysis or Kidney Transplantation Using Administrative Data **eTable 3.** Codes for Identifying Angiogram and Cardiac Catheterization Using Administrative Data **eTable 4.** Cohort Formation, Applying the >90 Days Chronicity Criterion **eTable 5.** Summary of Follow-up Times and Outcomes **eTable 6.** Incidence Rate Ratios (95% CIs) of Outpatient eGFR Measurements During Follow-up by Age **eTable 7.** Proportions and Outcomes of Cohort Members With a Single Outpatient eGFR Measurement During Follow-up **eTable 8.** Baseline Characteristics, by CKD Stage (Moving Average eGFR Method) **eFigure 1.** Study Design **eFigure 2.** Values, Counts, and Rates of eGFR Measurements During the Qualifying Period, by Age Categories **eFigure 3.** Number of Individuals, by Levels of Albuminuria and Age Categories **eFigure 4.** Cumulative Incidence Functions Over Time From Initial CKD Stage **eFigure 5.** Probabilities of Outcomes at 5 Years From Initial CKD Stage, by Levels of Albuminuria and Age Categories at Baseline **eFigure 6.** Probabilities of Outcomes at 5 Years From Initial CKD Stage, by Age Categories, in Those Without Inpatient or Emergency Department Serum Creatinine Measurements Within 3 Years Before Cohort Entry **eFigure 7.** Probabilities of Outcomes at 5 Years From Initial CKD Stage, by Age Categories, in Those Without Hospitalizations Within 3 Months Before Cohort Entry **eFigure 8.** Probabilities of Outcomes at 5 Years From Initial CKD Stage, by Age Categories, in Those Without Emergency Department Visits Within 3 Months Before Cohort Entry **eFigure 9.** Probabilities of Outcomes at 5 Years From Initial CKD Stage, by Age Categories, in Those Without Receiving Angiogram or Cardiac Catheterization Within 3 Months Before Cohort Entry **eFigure 10.** Probabilities of Outcomes at 5 Years From Initial CKD Stage, by Age Categories, in Those With No Events Within 1 Year or 2 Years **eFigure 11.** Probabilities of Outcomes at 5 Years From Initial CKD Stage, by Dispensation of ACEI/ARBs and Age Categories at Baseline **eFigure 12.** Probabilities of Outcomes at 5 Years From Initial CKD Stage, by Diabetes, Cardiovascular Disease, and Age Categories at Baseline **eFigure 13.** Cumulative Incidence Functions Over Time Following Regression **eFigure 14.** Probabilities of Outcomes at 5 Years Following Regression **eFigure 15.** Probabilities of Outcomes at 5 Years Following Regression, by Age Categories **eFigure 16.** Incidence of CKD, by CKD Stage and Age Categories (Moving Average eGFR Method) **eFigure 17.** Probabilities of Outcomes at 5 Years From Initial CKD Stage (Moving Average eGFR Method) **eFigure 18.** Probabilities of Outcomes at 5 Years From Initial CKD Stage, by Age Categories (Moving Average eGFR Method) **eFigure 19.** Probabilities of Outcomes at 5 Years From Initial CKD Stage (91-455 Days Chronicity Criterion) **eFigure 20.** Probabilities of Outcomes at 5 Years From Initial CKD Stage, by Age Categories (91-455 Days Chronicity Criterion) eReferences

## References

[1] United Nations, Department of Economic and Social Affairs, Population Division. World Population Ageing 2019. Published December 31, 2019. Accessed October 25, 2020. https://www.un.org/en/development/desa/population/publications/pdf/ageing/WorldPopulationAgeing2019-Report.pdf

[2] Gansevoort RT, Matsushita K, van der Velde M, ; Chronic Kidney Disease Prognosis Consortium. Lower estimated GFR and higher albuminuria are associated with adverse kidney outcomes: a collaborative meta-analysis of general and high-risk population cohorts. Kidney Int. 2011;80(1):93-104. doi:10.1038/ki.2010.531 21289597 PMC3959732

[3] Matsushita K, van der Velde M, Astor BC, ; Chronic Kidney Disease Prognosis Consortium. Association of estimated glomerular filtration rate and albuminuria with all-cause and cardiovascular mortality in general population cohorts: a collaborative meta-analysis. Lancet. 2010;375(9731):2073-2081. doi:10.1016/S0140-6736(10)60674-5 20483451 PMC3993088

[4] Ravani P, Quinn R, Fiocco M, . Association of age with risk of kidney failure in adults with stage IV chronic kidney disease in Canada. JAMA Netw Open. 2020;3(9):e2017150. doi:10.1001/jamanetworkopen.2020.17150 32945876 PMC7501537

[5] Jonsson AJ, Lund SH, Eriksen BO, Palsson R, Indridason OS. The prevalence of chronic kidney disease in Iceland according to KDIGO criteria and age-adapted estimated glomerular filtration rate thresholds. Kidney Int. 2020;98(5):1286-1295. doi:10.1016/j.kint.2020.06.017 32622831

[6] Bello AK, Levin A, Tonelli M, . Assessment of global kidney health care status. JAMA. 2017;317(18):1864-1881. doi:10.1001/jama.2017.4046 28430830 PMC5470418

[7] Kidney Disease: Improving Global Outcomes (KDIGO) CKD Work Group. KDIGO 2012 clinical practice guideline for the evaluation and management of chronic kidney disease. Kidney Int Suppl. 2013;3(1):1-150. doi:10.1038/kisup.2012.73

[8] Akbari A, Grimshaw J, Stacey D, . Change in appropriate referrals to nephrologists after the introduction of automatic reporting of the estimated glomerular filtration rate. CMAJ. 2012;184(5):E269-E276. doi:10.1503/cmaj.110678 22331970 PMC3307581

[9] Hemmelgarn BR, Zhang J, Manns BJ, ; Alberta Kidney Disease Network. Nephrology visits and health care resource use before and after reporting estimated glomerular filtration rate. JAMA. 2010;303(12):1151-1158. doi:10.1001/jama.2010.303 20332400

[10] Jain A, Hemmelgarn BR. Impact of estimated glomerular filtration rate reporting on nephrology referrals: a review of the literature. Curr Opin Nephrol Hypertens. 2011;20(3):218-223. doi:10.1097/MNH.0b013e3283446193 21490475

[11] Stevens KK, Woo YM, Rodger RS, Geddes CC. Discharging patients from the nephrology clinic to primary care—will they get appropriate monitoring of renal function? QJM. 2009;102(6):425-428. doi:10.1093/qjmed/hcp040 19376793

[12] Shardlow A, McIntyre NJ, Fluck RJ, McIntyre CW, Taal MW. Chronic kidney disease in primary care: outcomes after five years in a prospective cohort study. PLoS Med. 2016;13(9):e1002128. doi:10.1371/journal.pmed.1002128 27648564 PMC5029805

[13] Hu B, Gadegbeku C, Lipkowitz MS, ; African-American Study of Kidney Disease and Hypertension Group. Kidney function can improve in patients with hypertensive CKD. J Am Soc Nephrol. 2012;23(4):706-713. doi:10.1681/ASN.2011050456 22402803 PMC3312500

[14] Borrelli S, Leonardis D, Minutolo R, . Epidemiology of CKD regression in patients under nephrology care. PLoS One. 2015;10(10):e0140138. doi:10.1371/journal.pone.0140138 26462071 PMC4604085

[15] Fogo AB. Regression lines in chronic kidney disease. J Am Soc Nephrol. 2003;14(11):2990-2991. doi:10.1097/01.ASN.0000097862.14805.06 14569113

[16] Hemmelgarn BR, Clement F, Manns BJ, . Overview of the Alberta Kidney Disease Network. BMC Nephrol. 2009;10:30. doi:10.1186/1471-2369-10-30 19840369 PMC2770500

[17] Benchimol EI, Smeeth L, Guttmann A, ; RECORD Working Committee. The Reporting of Studies Conducted Using Observational Routinely-Collected Health Data (RECORD) statement. PLoS Med. 2015;12(10):e1001885. doi:10.1371/journal.pmed.1001885 26440803 PMC4595218

[18] Levey AS, Eckardt KU, Dorman NM, . Nomenclature for kidney function and disease: executive summary and glossary from a Kidney Disease: Improving Global Outcomes (KDIGO) consensus conference. Am J Kidney Dis. 2020;76(2):157-160. doi:10.1053/j.ajkd.2020.05.005 32565246

[19] Levey AS, Stevens LA, Schmid CH, ; CKD-EPI (Chronic Kidney Disease Epidemiology Collaboration). A new equation to estimate glomerular filtration rate. Ann Intern Med. 2009;150(9):604-612. doi:10.7326/0003-4819-150-9-200905050-00006 19414839 PMC2763564

[20] Statistics Canada. Census profile: 2016 census. Released November 29, 2017. Accessed February 25, 2020. https://www12.statcan.gc.ca/census-recensement/srvmsg/srvmsg404.html

[21] Barnett AG, van der Pols JC, Dobson AJ. Regression to the mean: what it is and how to deal with it. Int J Epidemiol. 2005;34(1):215-220. doi:10.1093/ije/dyh299 15333621

[22] Statistics Canada. Table 17-10-0005-01: population estimates on July 1st, by age and sex. Modified April 27, 2021. Accessed October 11, 2020. https://www150.statcan.gc.ca/t1/tbl1/en/tv.action?pid=1710000501

[23] Aalen OO, Johansen S. An empirical transition matrix for nonhomogeneous Markov chains based on censored observations. Scand Stat Theory Appl. 1978;5(3):141-150.

[24] Glassock RJ, Warnock DG, Delanaye P. The global burden of chronic kidney disease: estimates, variability and pitfalls. Nat Rev Nephrol. 2017;13(2):104-114. doi:10.1038/nrneph.2016.163 27941934

[25] von Scholten BJ, Persson F, Svane MS, Hansen TW, Madsbad S, Rossing P. Effect of large weight reductions on measured and estimated kidney function. BMC Nephrol. 2017;18(1):52. doi:10.1186/s12882-017-0474-0 28166744 PMC5294831

[26] Kennedy BK, Berger SL, Brunet A, . Geroscience: linking aging to chronic disease. Cell. 2014;159(4):709-713. doi:10.1016/j.cell.2014.10.039 25417146 PMC4852871

[27] Schiavon CC, Marchetti E, Gurgel LG, Busnello FM, Reppold CT. Optimism and hope in chronic disease: a systematic review. Front Psychol. 2017;7:2022. doi:10.3389/fpsyg.2016.02022 28101071 PMC5209342

