# Supplementary material for: Progression and Regression of Chronic Kidney Disease by Age Among Adults in a Population-Based Cohort in Alberta, Canada
Source: JAMA Netw Open. 2021 Jun 8;4(6):e2112828. doi: 10.1001/jamanetworkopen.2021.12828 (PMC8188272; doi:10.1001/jamanetworkopen.2021.12828)
Supplement: Supplement. — eMethods. CKD Cohorts, Average eGFR Method, and Covariates eTable 1. Methods for Cohort Formation and Outcome Definition eTable 2. Codes for Identifying Dialysis or Kidney Transplantation Using Administrative Data eTable 3. Codes for Identifying Angiogram and Cardiac Catheterization Using Administrative Data eTable 4. Cohort Formation, Applying the >90 Days Chronicity Criterion eTable 5. Summary of Follow-up Times and Outcomes eTable 6. Incidence Rate Ratios (95% CIs) of Outpatient eGFR Measurements During Follow-up by Age eTable 7. Proportions and Outcomes of Cohort Members With a Single Outpatient eGFR Measurement During Follow-up eTable 8. Baseline Characteristics, by CKD Stage (Moving Average eGFR Method) eFigure 1. Study Design eFigure 2. Values, Counts, and Rates of eGFR Measurements During the Qualifying Period, by Age Categories eFigure 3. Number of Individuals, by Levels of Albuminuria and Age Categories eFigure 4. Cumulative Incidence Functions Over Time From Initial CKD Stage eFigure 5. Probabilities of Outcomes at 5 Years From Initial CKD Stage, by Levels of Albuminuria and Age Categories at Baseline eFigure 6. Probabilities of Outcomes at 5 Years From Initial CKD Stage, by Age Categories, in Those Without Inpatient or Emergency Department Serum Creatinine Measurements Within 3 Years Before Cohort Entry eFigure 7. Probabilities of Outcomes at 5 Years From Initial CKD Stage, by Age Categories, in Those Without Hospitalizations Within 3 Months Before Cohort Entry eFigure 8. Probabilities of Outcomes at 5 Years From Initial CKD Stage, by Age Categories, in Those Without Emergency Department Visits Within 3 Months Before Cohort Entry eFigure 9. Probabilities of Outcomes at 5 Years From Initial CKD Stage, by Age Categories, in Those Without Receiving Angiogram or Cardiac Catheterization Within 3 Months Before Cohort Entry eFigure 10. Probabilities of Outcomes at 5 Years From Initial CKD Stage, by Age Categories, in Those With No Events Within 1 Year or 2 Year [file jamanetwopen-e2112828-s001.pdf]

Supplementary Online Content

Liu P, Quinn RR, Lam NN, et al. Progression and regression of chronic kidney disease by age among adults in a population-based cohort in Alberta, Canada. *JAMA Netw Open*. 2021;4(6):e2112828. doi:10.1001/jamanetworkopen.2021.12828

eMethods. CKD Cohorts, Mean eGFR Method, and Covariates
eTable 1. Methods for Cohort Formation and Outcome Definition
eTable 2. Codes for Identifying Dialysis or Kidney Transplantation Using Administrative Data
eTable 3. Codes for Identifying Angiogram and Cardiac Catheterization Using Administrative Data
eTable 4. Cohort Formation, Applying the >90 Days Chronicity Criterion
eTable 5. Summary of Follow-up Times and Outcomes
eTable 6. Incidence Rate Ratios (95% CIs) of Outpatient eGFR Measurements During Follow-up by Age
eTable 7. Proportions and Outcomes of Cohort Members With a Single Outpatient eGFR Measurement During Follow-up
eTable 8. Baseline Characteristics, by CKD Stage (Moving Average eGFR Method)
eFigure 1. Study Design
eFigure 2. Values, Counts, and Rates of eGFR Measurements During the Qualifying Period, by Age Categories
eFigure 3. Number of Individuals, by Levels of Albuminuria and Age Categories
eFigure 4. Cumulative Incidence Functions Over Time From Initial CKD Stage
eFigure 5. Probabilities of Outcomes at 5 Years From Initial CKD Stage, by Levels of Albuminuria and Age Categories at Baseline
eFigure 6. Probabilities of Outcomes at 5 Years From Initial CKD Stage, by Age Categories, in Those Without Inpatient or Emergency Department Serum Creatinine Measurements Within 3 Years Before Cohort Entry
eFigure 7. Probabilities of Outcomes at 5 Years From Initial CKD Stage, by Age Categories, in Those Without Hospitalizations Within 3 Months Before Cohort Entry
eFigure 8. Probabilities of Outcomes at 5 Years From Initial CKD Stage, by Age Categories, in Those Without Emergency Department Visits Within 3 Months Before Cohort Entry
eFigure 9. Probabilities of Outcomes at 5 Years From Initial CKD Stage, by Age Categories, in Those Without Receiving Angiogram or Cardiac Catheterization Within 3 Months Before Cohort Entry
eFigure 10. Probabilities of Outcomes at 5 Years From Initial CKD Stage, by Age Categories, in Those With No Events Within 1 Year or 2 Years
eFigure 11. Probabilities of Outcomes at 5 Years From Initial CKD Stage, by Dispensation of ACEI/ARBs and Age Categories at Baseline
eFigure 12. Probabilities of Outcomes at 5 Years From Initial CKD Stage, by Diabetes, Cardiovascular Disease, and Age Categories at Baseline
eFigure 13. Cumulative Incidence Functions Over Time Following Regression

© 2021 Liu P et al. *JAMA Network Open*.

eFigure 14. Probabilities of Outcomes at 5 Years Following Regression
eFigure 15. Probabilities of Outcomes at 5 Years Following Regression, by Age Categories
eFigure 16. Incidence of CKD, by CKD Stage and Age Categories (Moving Average eGFR Method)
eFigure 17. Probabilities of Outcomes at 5 Years From Initial CKD Stage (Moving Average eGFR Method)
eFigure 18. Probabilities of Outcomes at 5 Years From Initial CKD Stage, by Age Categories (Moving Average eGFR Method)
eFigure 19. Probabilities of Outcomes at 5 Years From Initial CKD Stage (91-455 Days Chronicity Criterion)
eFigure 20. Probabilities of Outcomes at 5 Years From Initial CKD Stage, by Age Categories (91-455 Days Chronicity Criterion)
eReferences

This supplementary material has been provided by the authors to give readers additional information about their work.

© 2021 Liu P et al. *JAMA Network Open*.

eMethods. CKD Cohorts, Mean eGFR Method, and Covariates

CKD cohorts

For each CKD cohort, we made efforts to minimize the inclusion of prevalent patients. First, individuals who were included in the mild CKD cohort were eligible to enter more severe CKD cohorts later during the study period. Individuals who were initially included in moderate CKD cohort could not enter the mild CKD cohort and those initially included in the severe CKD cohort were no longer eligible for inclusion in the mild-moderate CKD cohorts. Second, we used previous eGFR data (available from May 1, 2002) and kidney replacement data as look-back window and excluded: (1) those who had received dialysis or kidney transplantation on or prior to the index date; (2) those who had one or more outpatient eGFR <15 mL/min/1.73 m^2 prior to the qualifying period for each stage. Finally, since the index date had to fall between April 1, 2009 and March 31, 2015, we excluded those whose qualifying period for a CKD stage occurred before April 1, 2009 and qualified again on or after April 1, 2009 for the same or a less severe CKD stage. People who qualified to enter a stage prior to April 1, 2009 were eligible to enter the study on or after April 1, 2009 only if they qualified for a less severe CKD stage before April 1, 2009.

Identification of acute or chronic kidney replacement therapies: We used the provincial registration data (available from January 1, 2001) to identify chronic dialysis and kidney transplantation. Acute dialysis and early kidney transplantation that were not captured in the registration were identified from administrative data (available from April 1, 1994; related codes are shown in Appendix Table 2).

Moving average eGFR method

In the absence of a guideline on how to use routinely collected data to define a CKD stage and kidney outcomes,[1] in addition to the *sustained eGFR method* (main analyses), we implemented the *moving average eGFR method* (sensitivity analyses; Appendix Table 1). To select the earliest series (qualifying period) for cohort entry, the eGFR value defining the CKD stage was the mean (index eGFR) of all measurements in a qualifying period, and all eGFR values in that series of measurements were <60 mL/min/1.73 m^2. The date of the last eGFR in a qualifying period defined the index date (cohort entry). We also used the moving average eGFR method to ascertain kidney outcomes.

Covariates

We considered the following characteristics to describe the cohorts at baseline: age, sex, index eGFR, duration of the qualifying period, number of eGFR measurements during the qualifying period, prior outpatient eGFR (defined as the outpatient eGFR just prior to the qualifying period), albuminuria, comorbidities, acute conditions or receipt of potentially nephrotoxic procedures within 3 months prior to cohort entry (hospitalizations, emergency department visits, or receipt of angiogram or cardiac catheterization), and drugs dispensed within the year prior to cohort entry (Supplemental methods; eTable 3). We used the most recent outpatient albuminuria values before study entry and categorized as normal/mild, moderate, severe, or unmeasured, with the following types of measurement in descending order of preference: albumin-to-creatinine ratio (<30, 30 to 300, or >300 mg/g), protein-to-creatinine ratio (<150, 150 to 500, or >500 mg/g), and urine dipstick protein (negative or trace, 1+, or ≥2+).[1] If there were ≥2 same type measurements on the same day, we used the median value of ACR or PCR measurements; for urine dipstick measurements we applied floor function of median category that returns the largest integer less than or equal to a given value. We used validated algorithms[2] to identify important comorbidities, including diabetes, hypertension, cardiovascular disease (presence of congestive heart failure, myocardial infarction, peripheral vascular disease, or stroke), cancer (presence of metastatic cancer, non-metastatic cancer, or lymphoma), and dementia. We identified cohort members who had any hospitalizations, emergency department visits, or underwent angiogram or cardiac catheterization (code listed in eTable 3) within 3 months prior to cohort entry. We identified exposure to drugs including angiotensin-converting enzyme inhibitors or angiotensin receptor blockers (ACEI/ARBs), statins, and nonsteroidal anti-inflammatory drugs. This was defined as at least one dispensation for these drugs within the year before study entry, using provincial pharmaceutical databases.

© 2021 Liu P et al. *JAMA Network Open*.

eTable 1. Methods for Cohort Formation and Outcome Definition

1) Initial cohorts

Initial cohorts	Qualifying period for entry				Outcomes	Qualifying period for outcomes			
	First eGFR	Between	Last eGFR[a] *(Index date)*	Mean *eGFR*[b]		First eGFR	Between	Last eGFR[a] *(outcome date)*	Mean *eGFR*[b]
Sustained eGFR method									
Mild CKD	<60	<60	≥45 to <60	−	Regression	≥60	≥60	≥60	−
					Progression	<45	<45	<45	−
					Kidney failure$_{eGFR}$	<15	<15	<15	−
Moderate CKD	<45	<45	≥30 to <45	−	Regression	≥45	≥45	≥45	−
					Progression	<30	<30	<30	−
					Kidney failure$_{eGFR}$	<15	<15	<15	−
Severe CKD	<30	<30	≥15 to <30	−	Regression	≥30	≥30	≥30	−
					Kidney failure$_{eGFR}$	<15	<15	<15	−
Moving average eGFR method									
Mild CKD	<60	<60	<60	≥45 to <60	Regression	Any	Any	Any	≥60
					Progression	<60	<60	<60	<45
					Kidney failure$_{eGFR}$	<60	<60	<60	<15
Moderate CKD	<60	<60	<60	≥30 to <45	Regression	Any	Any	Any	≥45
					Progression	<60	<60	<60	<30
					Kidney failure$_{eGFR}$	<60	<60	<60	<15
Severe CKD	<60	<60	<60	≥15 to <30	Regression	Any	Any	Any	≥30
					Kidney failure$_{eGFR}$	<60	<60	<60	<15

© 2021 Liu P et al. *JAMA Network Open*.

2) Sub-cohorts following regression

**Initial cohorts**	**Sub-cohort regressed to**	**Outcomes**	**Qualifying period for stage change**			
			First eGFR	Between	Last eGFR[c] *(outcome date)*	Mean *eGFR*[d]
Sustained eGFR method						
Mild CKD	G1-2	Progression	<60	<60	<60	−
		Kidney failure$_{eGFR}$	<15	<15	<15	−
Moderate CKD	G1-3a	Further regression	≥60	≥60	≥60	−
		Progression	<45	<45	<45	−
		Kidney failure$_{eGFR}$	<15	<15	<15	−
Severe CKD	G1-3b	Further regression	≥45	≥45	≥45	−
		Progression	<30	<30	<30	−
		Kidney failure$_{eGFR}$	<15	<15	<15	−
Moving average eGFR method						
Mild CKD	G1-2	Progression	<60	<60	<60	<60
		Kidney failure$_{eGFR}$	<60	<60	<60	<15
Moderate CKD	G1-3a	Further regression	Any	Any	Any	≥60
		Progression	<60	<60	<60	<45
		Kidney failure$_{eGFR}$	<60	<60	<60	<15
Severe CKD	G1-3b	Further regression	Any	Any	Any	≥45
		Progression	<60	<60	<60	<30
		Kidney failure$_{eGFR}$	<60	<60	<60	<15

Note: eGFR, estimated glomerular filtration rate (mL/min/1.73 m^2).

© 2021 Liu P et al. *JAMA Network Open*.

[a] For the initial cohort using the sustained method, the last eGFR during the qualifying period for cohort entry was the baseline eGFR. Outcome of regression was defined as a sustained improvement to a better eGFR category for >3 months, accompanied by a 25% or greater increase in the last eGFR from baseline; Outcome of progression is defined as a sustained drop in eGFR category for >3 months, accompanied by a 25% or greater drop in the last eGFR from baseline.

[b] For the initial cohort using the moving average method, the mean eGFR during the qualifying period for cohort entry was the baseline eGFR. Outcome of regression was defined as an improvement to a better eGFR category accompanied by a 25% or greater increase in the mean eGFR from baseline; Outcome of progression is defined as a drop in eGFR category accompanied by a 25% or greater drop in the mean eGFR from baseline.

[c] For the sub-cohort following regression using the sustained method, the new baseline eGFR (the last eGFR) was updated at the time of regression. Outcome of further regression was defined as a sustained improvement to a better eGFR category for >3 months, accompanied by a 25% or greater increase in the last eGFR from the new baseline; Outcome of progression is defined as a sustained drop in eGFR category for >3 months, accompanied by a 25% or greater drop in the last eGFR from the new baseline.

[d] For the sub-cohort following regression using the moving average method, the new baseline eGFR (the mean eGFR) was updated at the time of regression. Outcome of regression was defined as an improvement to a better eGFR category accompanied by a 25% or greater increase in the mean eGFR from the new baseline; Outcome of progression is defined as a drop in eGFR category accompanied by a 25% or greater drop in the mean eGFR from the new baseline.

© 2021 Liu P et al. *JAMA Network Open*.

eTable 2. Codes for Identifying Dialysis or Kidney Transplantation Using Administrative Data

1) Physician claims: Canadian Classification of Diagnostic, Therapeutic, and Surgical Procedures codes

Codes	Code description
For dialysis	
13.99A	Hemodialysis treatment, unstable patient
13.99B	Hemodialysis treatment, stable patient
13.99C	Assessment and management of an unstable patient with acute/chronic renal failure treated by peritoneal dialysis
13.99D	Assessment and management of a stable patient with chronic renal failure treated by peritoneal dialysis
13.99O	Management of dialysis patients on home dialysis or receiving treatment in a remote hemodialysis unit (per week)
13.99OA	Management of patient on hemodialysis or peritoneal dialysis (per week)
13.99AB	Dialysis therapy, any modality, in the intensive care unit
For transplantation	
67.5	Transplant of kidney
67.59	Other kidney transplantation
67.59A	Renal transplantation (homo, hetero, auto)

2) Hospitalizations

Codes	Code description
For transplantation: Canadian Classification of Health Intervention codes	
1.PC.85.^^	Transplant, kidney
1.PC.85.LA-XX-J	Using living donor (allogenic or syngeneic) kidney
1.PC.85.LA-XX-K	Using deceased donor kidney
1.OK.85.XU-XX-K	Transplant, pancreas with duodenum and kidney with exocrine drainage via bladder [e.g. donor duodenum is grafted to bladder: duodenocystostomy]
1.OK.85.XV-XX-K	Transplant, pancreas with duodenum and kidney with exocrine drainage via intestine with homograft [e.g. donor duodenum is grafted to bowel]
For transplantation: ICD-9-CM procedure codes	
55.69	Other kidney transplantation

© 2021 Liu P et al. *JAMA Network Open*.

eTable 3. Codes for Identifying Angiogram and Cardiac Catheterization Using Administrative Data

Codes	Code description
Hospitalizations or Ambulatory Care Classification System (ACCS): Canadian Classification of Health Intervention (CCI) codes	
3.IP.10.^^	**Xray, heart with coronary arteries**<br>***Includes:*** Angiocardiography involving heart and coronary arteries<br>Angiography, coronary<br>Cardiac catheterization for coronary angiography<br>Coronary arteriography<br>Ventriculography with coronary angiograms<br>Xray with fluoroscopy, heart
3.ID.10.^^	**Xray, aorta** not elsewhere classified<br>***Includes:*** Angiography, aorta [ascending, thoracic, abdominal, arch]<br>Angiography, combined abdominal with thoracic aorta<br>Aortic root angiogram<br>Aortography, NEC<br>xray with fluoroscopy, aorta not elsewhere classified
3.IS.10.VC	**Xray, vena cava** (superior and/or inferior) following intravenous injection of contrast (with or without fluoroscopy)<br>***Includes:*** Phlebography, vena cava<br>Venacavogram<br>Venography, vena cava<br>Xray with fluoroscopy, vena cava
Physician claims: Canadian Classification of Diagnostic, Therapeutic, and Surgical Procedures (CCPx) Codes	
47.72C	Percutaneous closure, atrial septal defect
48.92A	Selective angiocardiogram
48.93	Angiocardiography of right heart structures
48.94	Angiocardiography of left heart structures
48.95	Combined right and left heart angiocardiography
48.96	Coronary arteriography using a single catheter
48.97	Coronary arteriography using two catheters
48.98	Other coronary arteriography
49.95	Right cardiac catheterization
49.96	Left cardiac catheterization
50.71C	Balloon embolization of caroticocavernous fistula Includes intraoperative angiograms
50.79A	Vascular occlusion by catheter, to include intraoperative angiograms, any area
50.8	Selective angiography using contrast material
51.59A	Open transluminal angioplasty
51.59B	Percutaneous transluminal angioplasty, excluding coronary vessels
51.59D	Percutaneous transluminal coronary angioplasty with associated diagnostic angiogram
51.99B	Percutaneous removal or lysis of embolus or thrombus in any vessel

© 2021 Liu P et al. *JAMA Network Open*.

eTable 4. Cohort Formation, Applying the >90 Days Chronicity Criterion

Number of residents registered in Alberta Health Apr 1, 1994 – Mar 31, 2017		4,983,956	
**Common exclusion criteria (N excluded):**		4,644,966	
• No serum creatinine measurements (available May 1, 2002 – Mar 31, 2017)		1,573,084	
• No outpatient serum creatinine measurements		201,969	
• All outpatient serum creatinine were tested under 18 years old		31,971	
• All outpatient serum creatinine measurements were tested after the earliest of death, out-migration, registration end or accrual end (March 31, 2017)		6,099	
• Never had an outpatient eGFR measurement <60 mL/min/1.73 m^2		2,697,464	
• Only 1 outpatient eGFR <60 mL/min/1.73 m^2		129,412	
• The first eGFR less than 60 was already <15 mL/min/1.73 m^2		4,967	
**CKD stage**	**Mild CKD**	**Moderate CKD**	**Severe CKD**
**Unique exclusion criteria (N excluded):**	257,670	303,061	326,753
• Did not meet the sustained eGFR criterion for a specific stage, for >90 d	130,257	246,755	307,004
• Index date was not between Apr 1, 2009 and Mar 31, 2015	126,787	55,644	19,030
• Index date was on the earliest date of death, out-migration, registration end or accrual end	72	21	10
• At least 1 outpatient eGFR <15 mL/min/1.73 m^2 prior to the qualifying period	83	193	342
• Initiated kidney replacement therapy on or prior to index date	471	448	367
**Cohort size (N)**	**81,320**	**35,929**	**12,237**

Note:

1) Our population registry started in 1994. Serum creatinine data started on May 1, 2002, with nearly complete coverage (~98% of the Alberta population) from July 1, 2003 and complete coverage from January 1, 2005. The ~1/3 of the population (n=1,573,084) who never had an eGFR measurement largely comprises people who were in the registry before the study start date (April 1, 2009). We considered all the available information preceding Apr 1, 2009 (look-back window for eGFR measurements and other data) to minimize the inclusion of prevalent cases in the incident CKD cohorts between Apr 1, 2009 and Mar 31, 2015.
2) Of those who were registered for at least 1 fiscal year between Apr 1, 2009 and Mar 31, 2015, 20% did not have a serum creatinine measurement. Most of these (~85%) were younger than 40 years, an expected figure considering that a screening test for serum creatinine wouldn't normally be recommended for most people under 40 unless they are at increased risk of developing CKD.

© 2021 Liu P et al. *JAMA Network Open*.

eTable 5. Summary of Follow-up Times and Outcomes

CKD stage	Mild CKD	Moderate CKD	Severe CKD
**Initial cohort**			
N	81,320	35,929	12,237
Follow-up years, median (IQR)	3.44 (2.25−5.00)	2.80 (1.59−4.34)	2.15 (0.99−3.48)
Outcomes, n (%)			
Censored	51,205 (63.0)	16,422 (45.7)	3,635 (29.7)
Regression	10,052 (12.4)	6,021 (16.8)	2,173 (17.8)
Progression	9,605 (11.8)	4,895 (13.6)	-
Kidney failure (eGFR)	6 (0.01)	30 (0.08)	1,818 (14.9)
Kidney failure (kidney replacement)	28 (0.03)	72 (0.20)	370 (3.0)
Death	10,424 (12.8)	8,489 (23.6)	4,241 (34.7)
**Sub-cohort following regression**			
N	10,052	6,021	2,173
Follow-up years, median (IQR)	1.90 (0.96−3.29)	1.77 (0.88−3.04)	1.65 (0.85−2.83)
Outcomes, n (%)			
Censored	6,775 (67.4)	3,360 (55.8)	1,042 (48.0)
Further regression	-	236 (3.9)	97 (4.5)
Progression	1,564 (15.6)	1,102 (18.3)	409 (18.8)
Kidney failure (eGFR)	0	0	1 (0.05)
Kidney failure (kidney replacement)	1 (0.01)	0	0
Death	1,712 (17.0)	1,323 (22.0)	624 (28.7)

© 2021 Liu P et al. *JAMA Network Open*.

eTable 6. Incidence Rate Ratios (95% CIs) of Outpatient eGFR Measurements During Follow-up by Age

CKD stage	Mild CKD	Moderate CKD	Severe CKD
Baseline incidence rate, per person-year	3.00 (2.97−3.04)	5.69 (5.55−5.82)	9.30 (8.99−9.61)
Age categories, years			
18-64	Reference	Reference	Reference
65-74	0.92 (0.91−0.94)	0.85 (0.83−0.88)	0.91 (0.87−0.95)
75-84	0.89 (0.88−0.91)	0.74 (0.72−0.76)	0.80 (0.77−0.83)
≥85	0.88 (0.86−0.90)	0.64 (0.62−0.66)	0.64 (0.61−0.67)

Estimates were derived from negative binomial regression.

© 2021 Liu P et al. *JAMA Network Open*.

eTable 7. Proportions and Outcomes of Cohort Members With a Single Outpatient eGFR Measurement During Follow-up

**Initial cohort**	**Mild CKD**		**Moderate CKD**		**Severe CKD**	
	All	Age ≥65 y	All	Age ≥65 y	All	Age ≥65 y
N	81 320	61 646	35 929	30 998	12 237	10 041
Only had 1 follow-up eGFR	4891 (6.0)	3783 (6.1)	1704 (4.7)	1592 (5.1)	555 (4.5)	531 (5.3)
Outcomes						
Censored	3358 (68.7)	2366 (62.5)	680 (39.9)	605 (38.0)	100 (18.0)	88 (16.6)
Regression	-	-	-	-	-	-
Progression	-	-	-	-	-	-
Kidney failure	-	-	-	-	-	-
Death	1533 (31.3)	1417 (37.5)	1024 (60.1)	987 (62.0)	455 (82.0)	443 (83.4)
The follow-up eGFR:						
Same eGFR category	2806 (57.4)	2198 (58.1)	420 (24.6)	379 (23.8)	4 (0.7)	4 (0.8)
Drop in eGFR category	603 (12.3)	537 (14.2)	1232 (72.3)	1167 (73.3)	547 (98.6)	524 (98.7)
Increase in eGFR category	1482 (30.3)	1048 (27.7)	52 (3.1)	46 (2.9)	4 (0.7)	3 (0.6)
The follow-up eGFR:						
Drop in eGFR category + 25% decrease from baseline	4	4	8	6	4	4
Increase in eGFR category + 25% increase from baseline	451	331	52	46	4	3

Values are presented in number or number (percent).

© 2021 Liu P et al. *JAMA Network Open*.

eTable 8. Baseline Characteristics, by CKD Stage (Moving Average eGFR Method)

Cohorts	Mild CKD (N=81,182)	Moderate CKD (N=48,166)	Severe CKD (N=17,555)
Age, in years, mean (SD)	72.4 (11.3)	76.3 (11.8)	76.7 (13.6)
18-64	19,674 (24.2)	7,561 (15.7)	3,046 (17.4)
65-74	25,937 (32.0)	11,638 (24.2)	3,312 (18.9)
75-84	25,402 (31.3)	17,621 (36.6)	5,979 (34.1)
≥85	10,169 (12.5)	11,346 (23.6)	5,218 (29.7)
Men	36,422 (44.9)	21,443 (44.5)	8,036 (45.8)
**Kidney health measures**<br>Index eGFR, mean (SD)	53.8 (3.6)	41.7 (3.2)	27.5 (2.7)
Qualifying period, days, median (IQR)	266 (149 − 427)	197 (121 − 369)	144 (105 − 242)
≤365	53,235 (65.6)	35,896 (74.5)	15,194 (86.6)
>365 to ≤455	14,509 (17.9)	6,205 (12.9)	1,214 (6.9)
>455 to ≤730	5,402 (6.7)	2,362 (4.9)	500 (2.8)
>730	8,036 (9.9)	3,703 (7.7)	647 (3.7)
Qualifying period, number of outpatient eGFR measurements, mean (SD)	2 (1)	3 (2)	4 (2)
Prior outpatient eGFR<br>Unmeasured	6,924 (8.5)	3,136 (6.5)	692 (3.9)
≥60	74,258 (91.5)	11,298 (23.5)	893 (5.1)
≥45 to <60	0	32,344 (67.2)	2,534 (14.4)
≥30 to <45	0	1,387 (2.9)	13,020 (74.2)
≥15 to <30	0	1 (0)	416 (2.4)
Albuminuria[a]<br>Unmeasured	4,197 (5.2)	2,311 (4.8)	626 (3.6)
Normal or mild	63,030 (77.6)	31,831 (66.1)	8,340 (47.5)
Moderate	9,864 (12.2)	9,005 (18.7)	4,397 (25.1)
Severe	4,091 (5.0)	5,019 (10.4)	4,192 (23.9)
**Comorbidities**<br>Diabetes	23,619 (29.1)	18,648 (38.7)	8,568 (48.8)
Hypertension	62,430 (76.9)	42,647 (88.5)	16,288 (92.8)
Cardiovascular disease	25,042 (30.8)	22,025 (45.7)	10,082 (57.4)
Congestive heart failure	11,775 (14.5)	12,909 (26.8)	7,037 (40.1)
Myocardial infarction	5,176 (6.4)	4,577 (9.5)	2,061 (11.7)
Peripheral vascular disease	2,925 (3.6)	2,856 (5.9)	1,394 (7.9)
Stroke or TIA	12,662 (15.6)	10,541 (21.9)	4,585 (26.1)
Cancer	10,994 (13.5)	7,912 (16.4)	3,154 (18.0)
Lymphoma	1,047 (1.3)	926 (1.9)	447 (2.5)
Metastatic	2,530 (3.1)	2,050 (4.3)	838 (4.8)
Non-metastatic	9,426 (11.6)	6,623 (13.8)	2,564 (14.6)
Dementia	5,710 (7.0)	5,566 (11.6)	2,566 (14.6)

© 2021 Liu P et al. *JAMA Network Open*.

**Indicators of acute conditions**			
Hospitalization	4,793 (5.9)	5,921 (12.3)	3,350 (19.1)
Emergency department visit	10,832 (13.3)	10,193 (21.2)	4,928 (28.1)
Receipt of angiogram or cardiac catheterization	594 (0.7)	648 (1.3)	225 (1.3)
**Drugs dispensed**			
ACEI or ARBs	49,540 (61.0)	36,008 (74.8)	13,906 (79.2)
Statins	34,294 (42.2)	23,280 (48.3)	9,339 (53.2)
NSAIDs	17,998 (22.2)	10,846 (22.5)	3,005 (17.1)

Abbreviations: ACEI/ARBs, angiotensin-converting enzyme inhibitor or angiotensin receptor blockers; CKD, chronic kidney disease; eGFR, estimated glomerular filtration rate (mL/min/1.73 m^2); IQR, inter quartile range; NSAIDs, nonsteroidal anti-inflammatory drugs; SD, standard deviation; TIA, transient ischemic attack.
Values are number (%), otherwise stated.
[a]Albuminuria was categorized as normal/mild, moderate, severe or unmeasured, based on the most recent outpatient values, with the following types of measurement in descending order of preference: albumin-to-creatinine ratio (<30, 30 to 300, or >300 mg/g), protein-to-creatinine ratio (<150, 150 to 500, or >500 mg/g), and urine dipstick (negative or trace, 1+, or ≥2+).

© 2021 Liu P et al. *JAMA Network Open*.

eFigure 1. Study Design

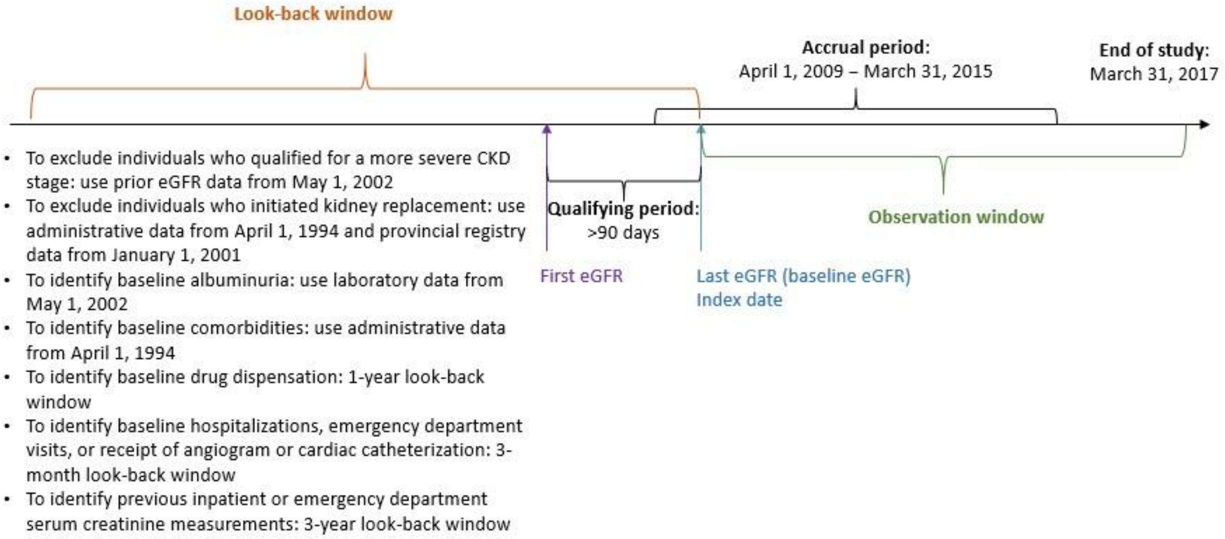

© 2021 Liu P et al. *JAMA Network Open*.

eFigure 2. Values, Counts, and Rates of eGFR Measurements During the Qualifying Period, by Age Categories

A) Values

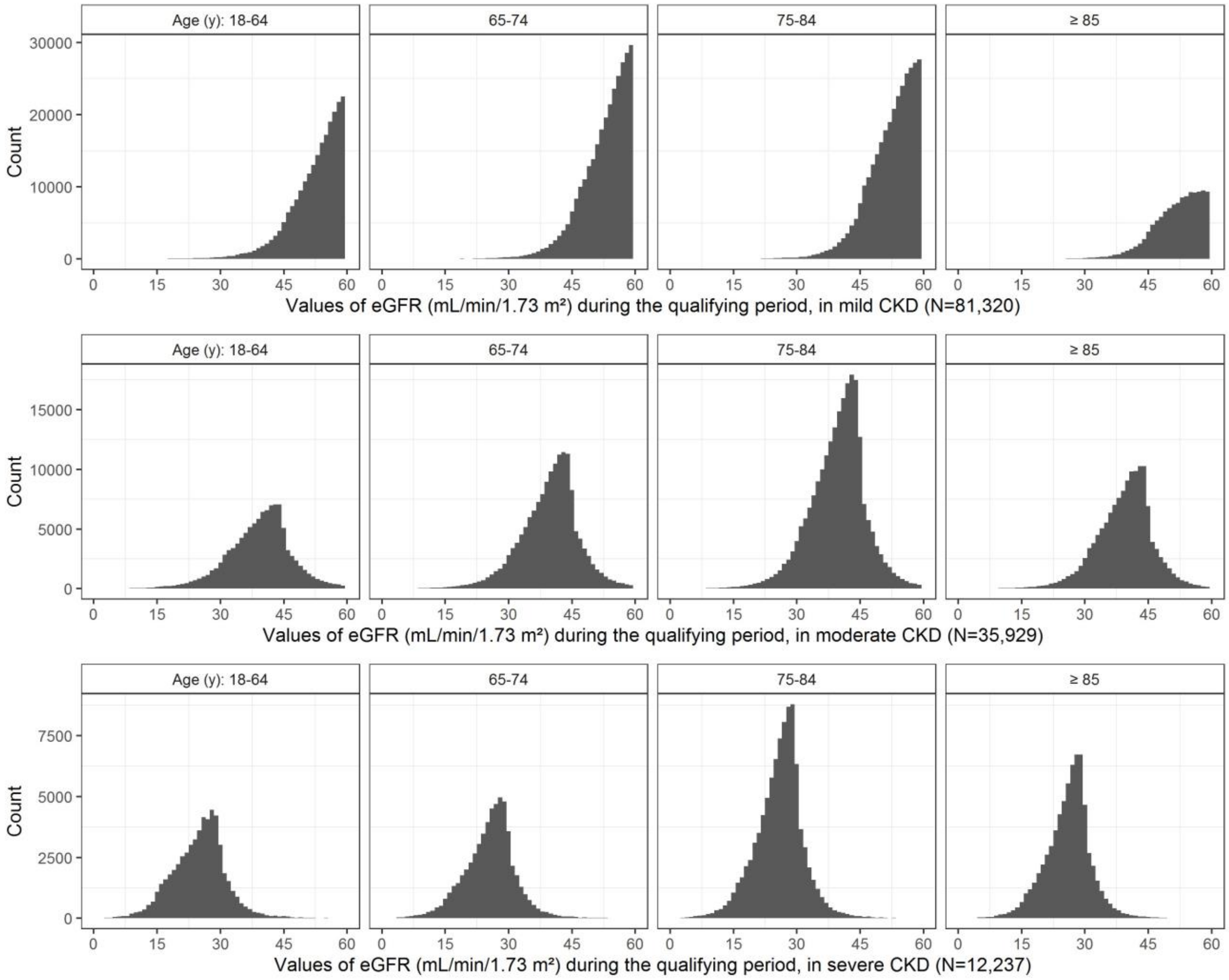

© 2021 Liu P et al. *JAMA Network Open*.

B) Counts

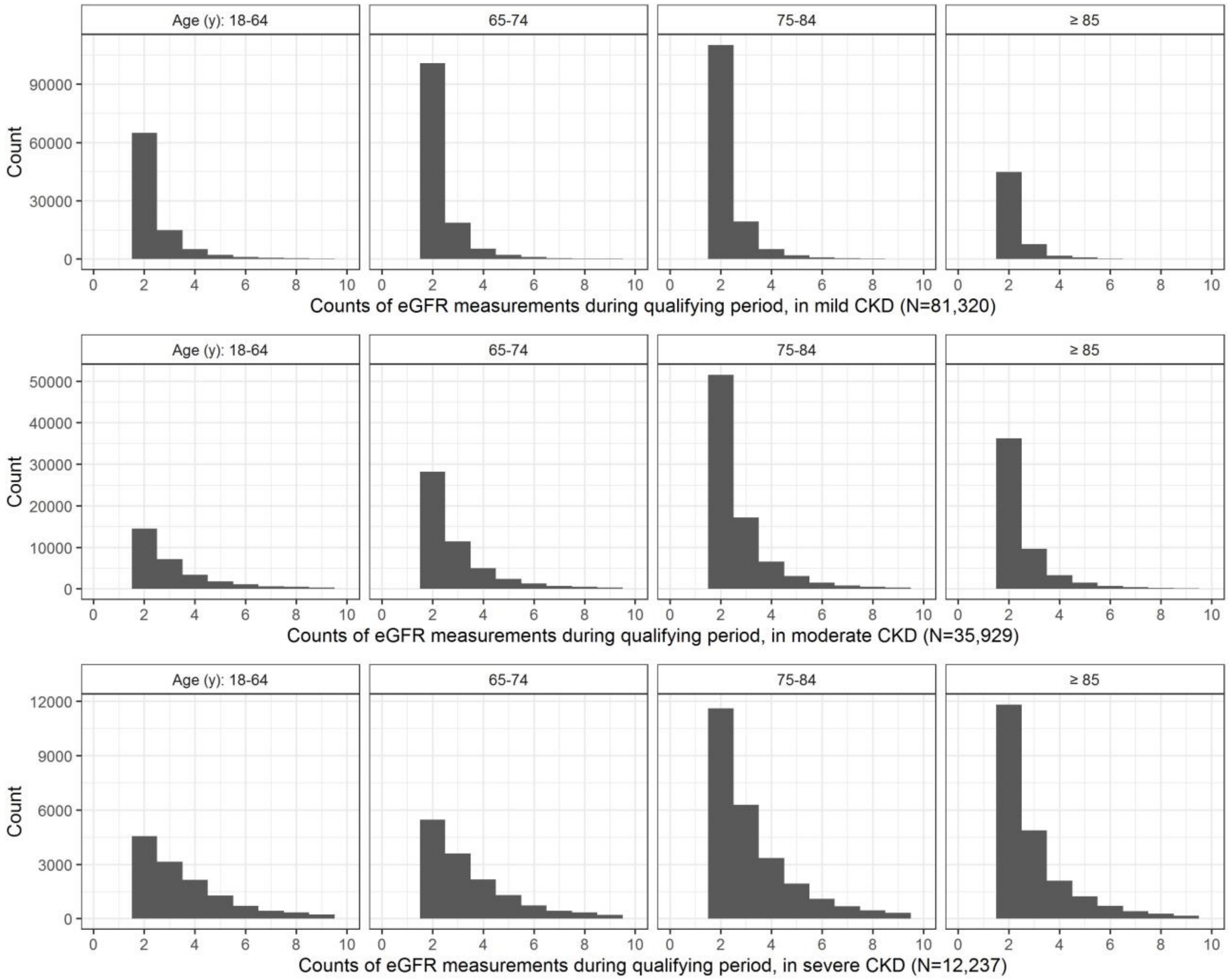

© 2021 Liu P et al. *JAMA Network Open*.

C) Rates

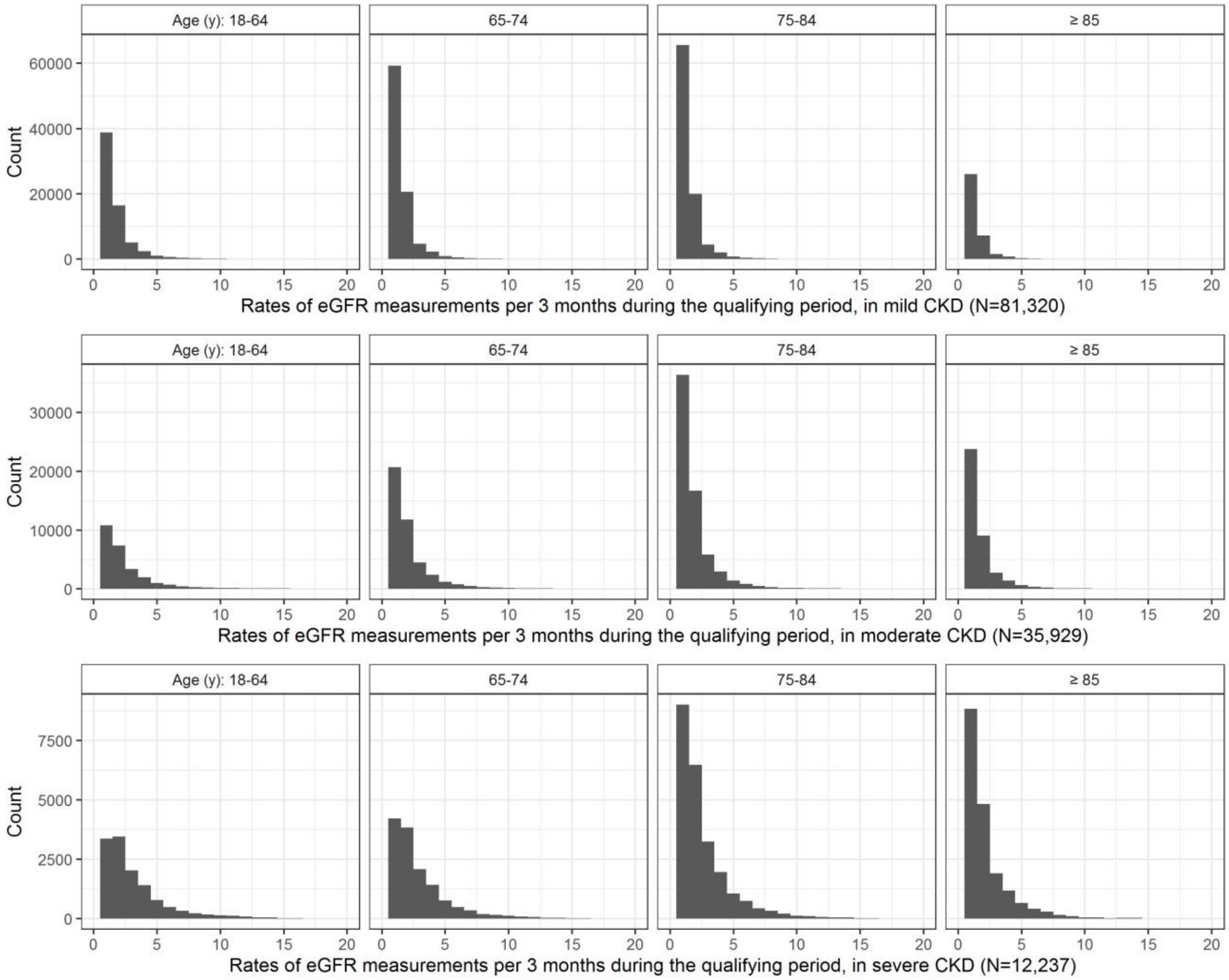

© 2021 Liu P et al. *JAMA Network Open*.

eFigure 3. Number of Individuals, by Levels of Albuminuria and Age Categories

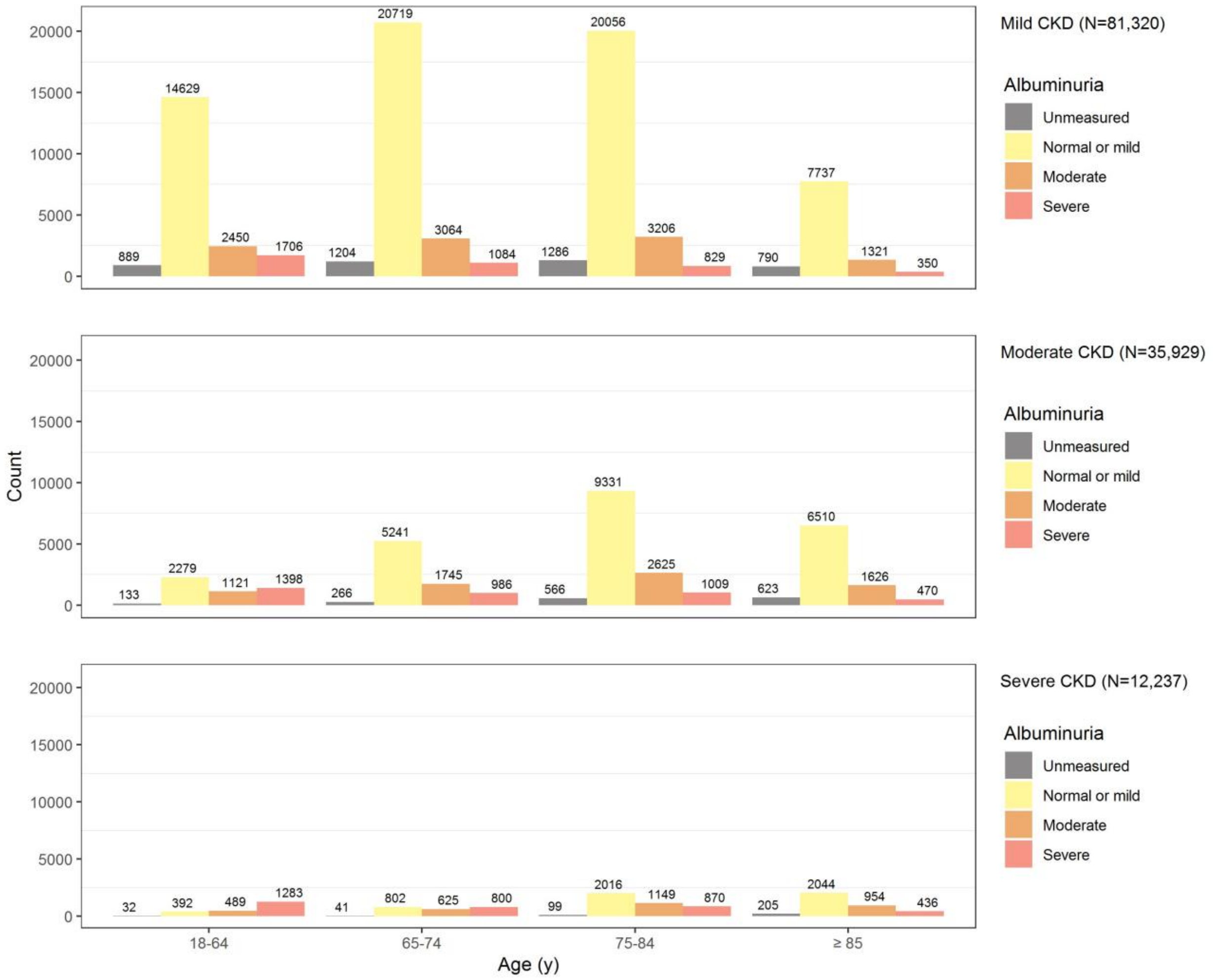

Legend: Albuminuria was categorized as normal/mild, moderate, severe or unmeasured, based on the most recent outpatient values, with the following types of measurement in descending order of preference: albumin-to-creatinine ratio (<30, 30 to 300, or >300 mg/g), protein-to-creatinine ratio (<150, 150 to 500, or >500 mg/g), and urine dipstick (negative or trace, 1+, or ≥2+).

© 2021 Liu P et al. *JAMA Network Open*.

eFigure 4. Cumulative Incidence Functions Over Time From Initial CKD Stage

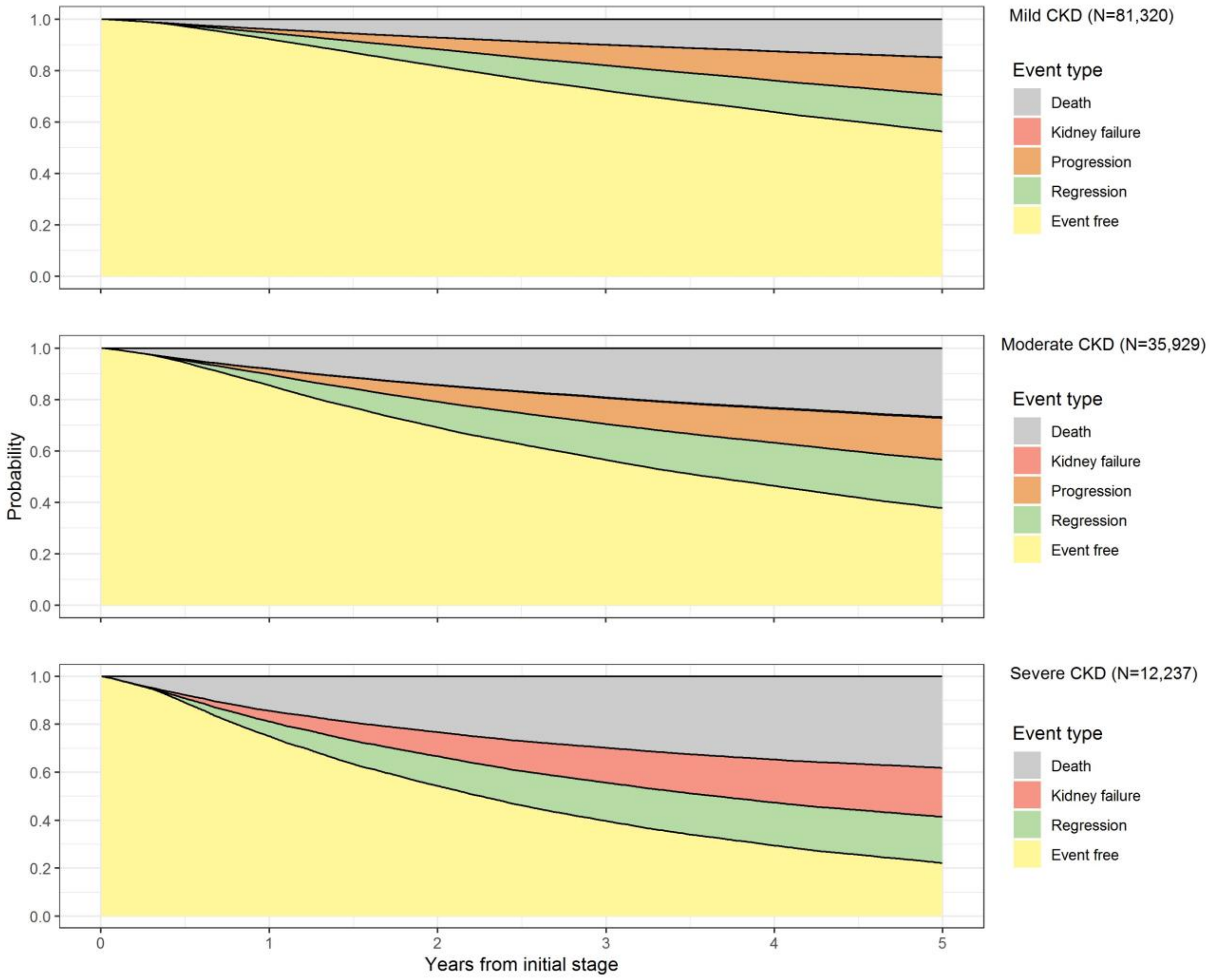

© 2021 Liu P et al. *JAMA Network Open*.

eFigure 5. Probabilities of Outcomes at 5 Years From Initial CKD Stage, by Levels of Albuminuria and Age Categories at Baseline

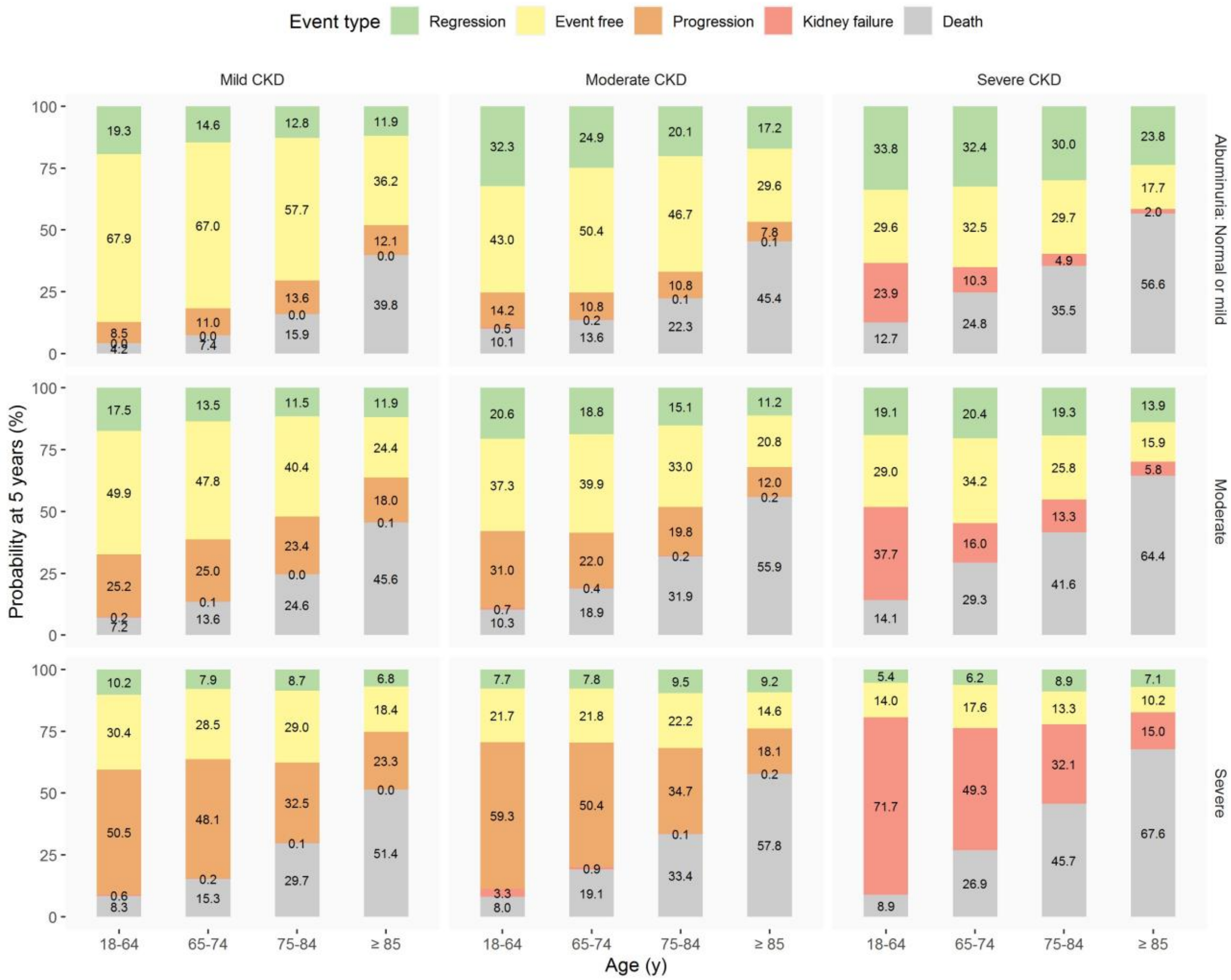

Legend: Albuminuria was categorized as normal/mild, moderate, or severe, based on the most recent outpatient values, with the following types of measurement in descending order of preference: albumin-to-creatinine ratio (<30, 30 to 300, or >300 mg/g), protein-to-creatinine ratio (<150, 150 to 500, or >500 mg/g), and urine dipstick (negative or trace, 1+, or ≥2+).

© 2021 Liu P et al. *JAMA Network Open*.

eFigure 6. Probabilities of Outcomes at 5 Years From Initial CKD Stage, by Age Categories, in Those Without Inpatient or Emergency Department Serum Creatinine Measurements Within 3 Years Before Cohort Entry

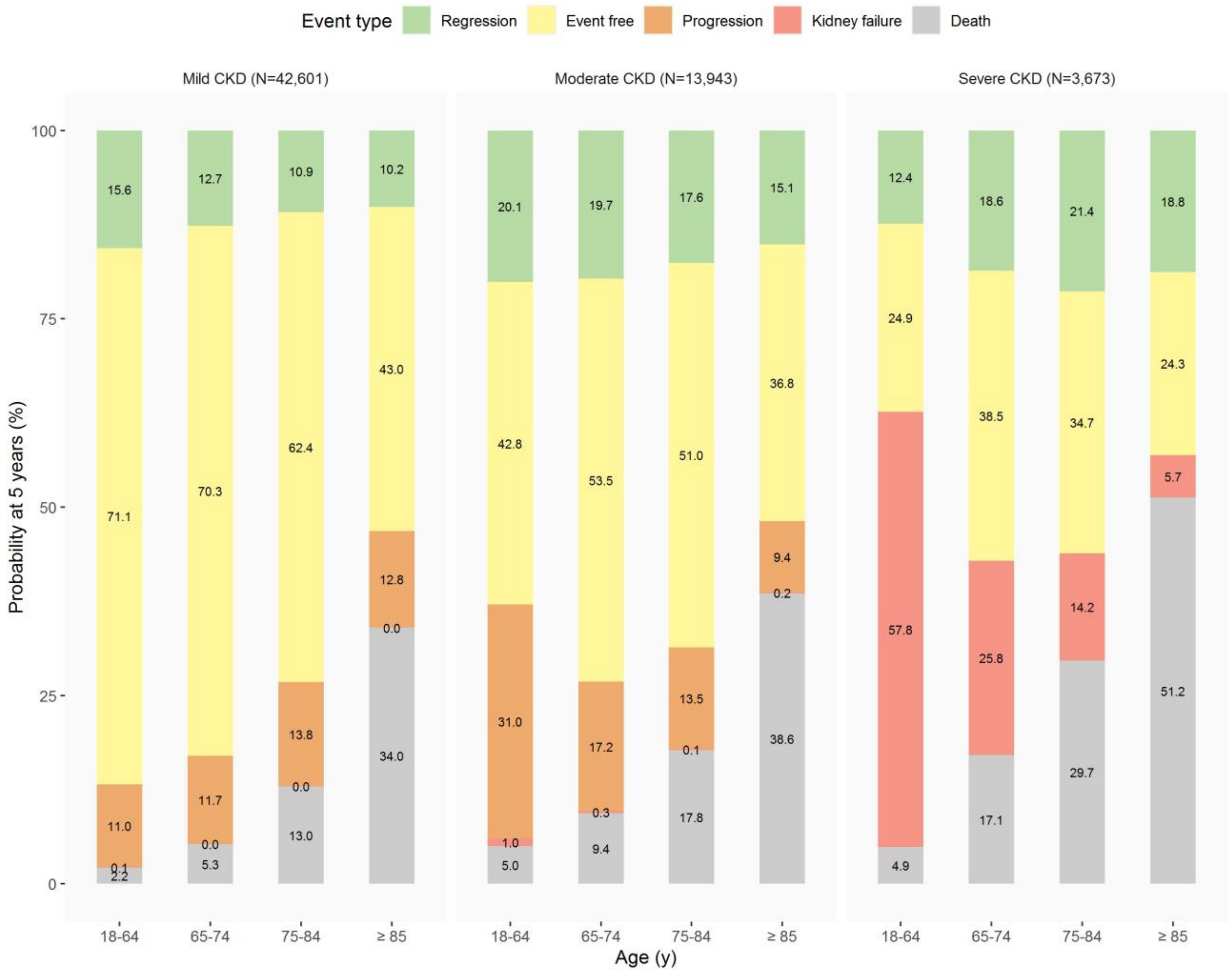

© 2021 Liu P et al. *JAMA Network Open*.

eFigure 7. Probabilities of Outcomes at 5 Years From Initial CKD Stage, by Age Categories, in Those Without Hospitalizations Within 3 Months Before Cohort Entry

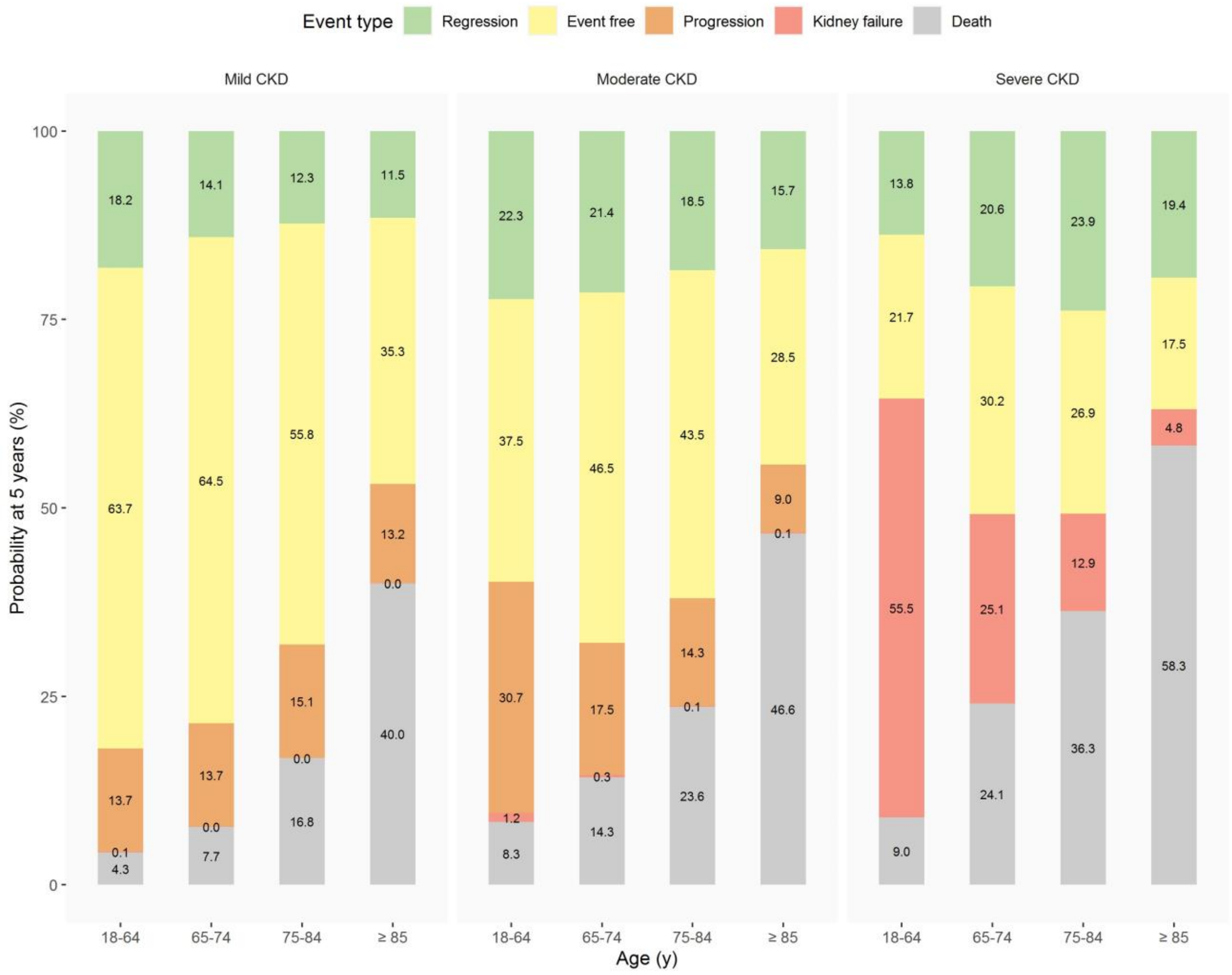

© 2021 Liu P et al. *JAMA Network Open*.

eFigure 8. Probabilities of Outcomes at 5 Years From Initial CKD Stage, by Age Categories, in Those Without Emergency Department Visits Within 3 Months Before Cohort Entry

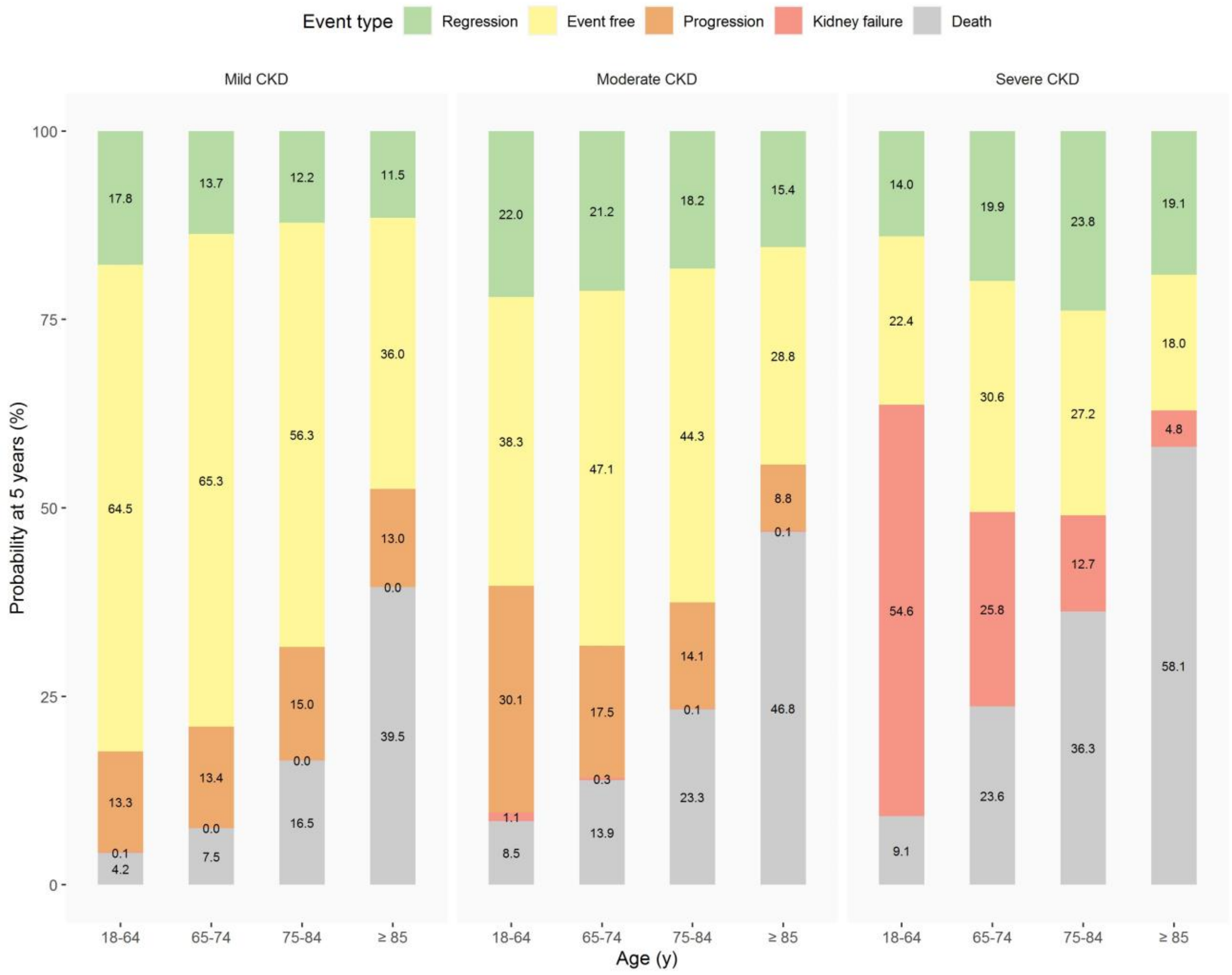

© 2021 Liu P et al. *JAMA Network Open*.

eFigure 9. Probabilities of Outcomes at 5 Years From Initial CKD Stage, by Age Categories, in Those Without Receiving Angiogram or Cardiac Catheterization Within 3 Months Before Cohort Entry

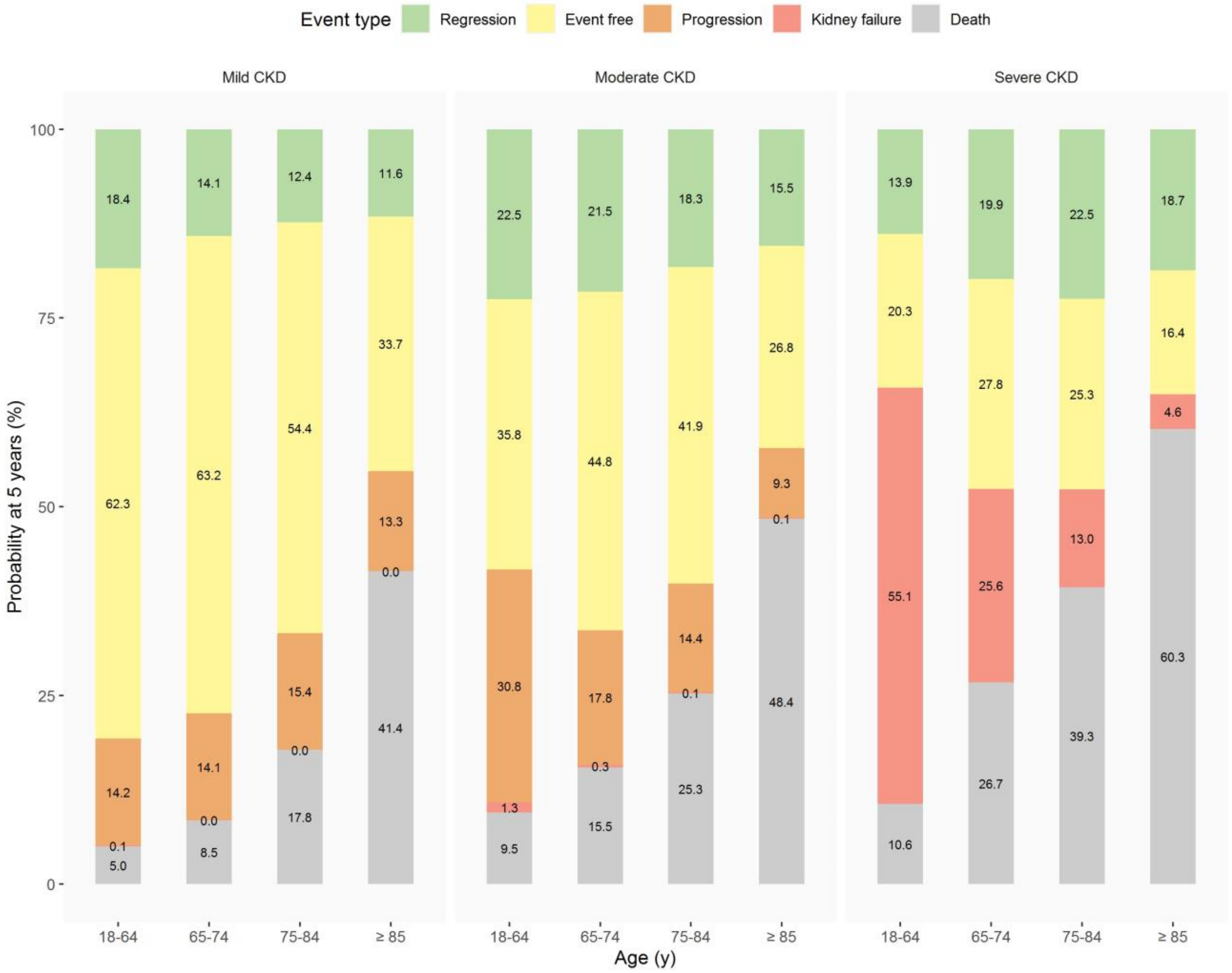

© 2021 Liu P et al. *JAMA Network Open*.

eFigure 10. Probabilities of Outcomes at 5 Years From Initial CKD Stage, by Age Categories, in Those With No Events Within 1 Year or 2 Years

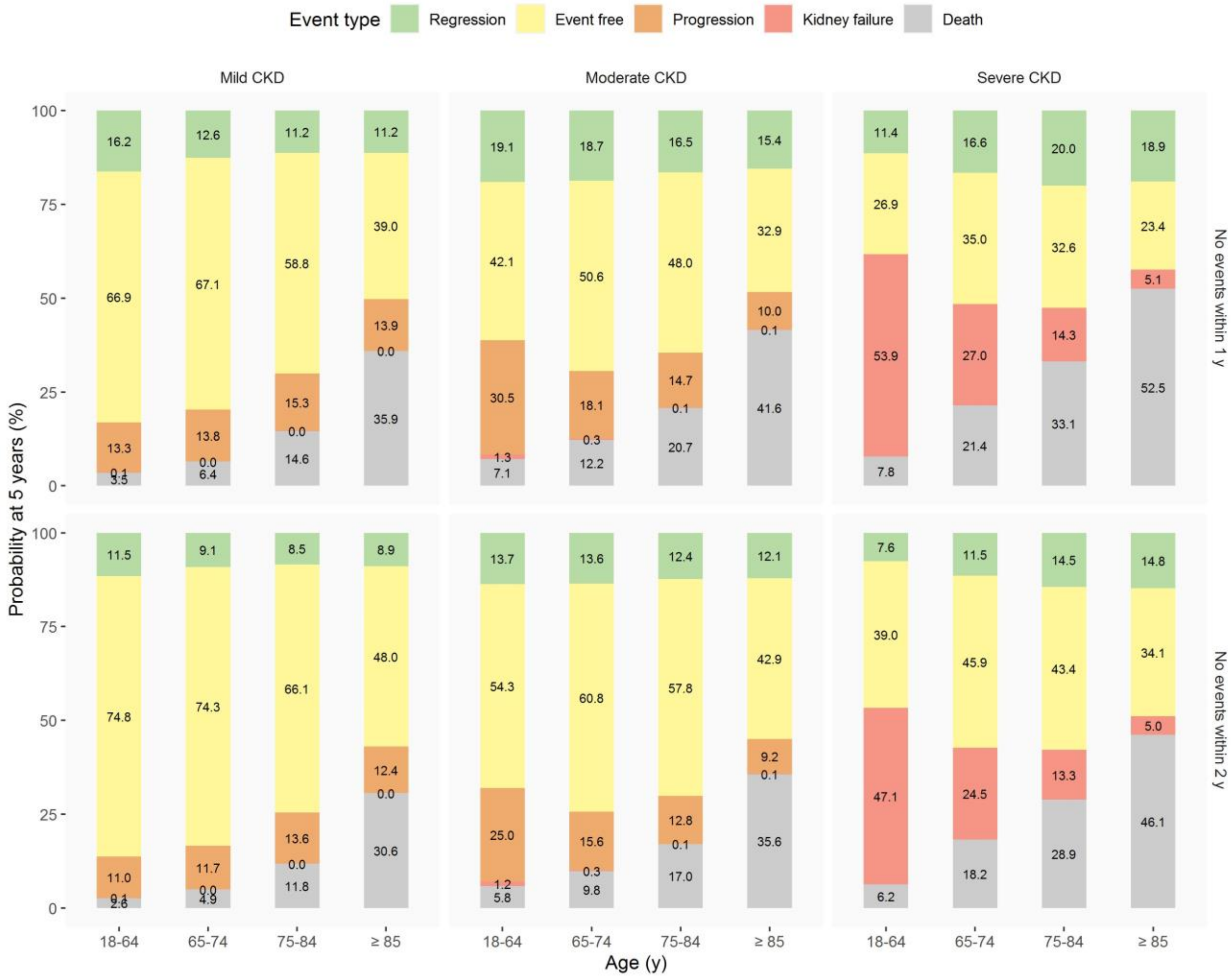

© 2021 Liu P et al. *JAMA Network Open*.

eFigure 11. Probabilities of Outcomes at 5 Years From Initial CKD Stage, by Dispensation of ACEI/ARBs and Age Categories at Baseline

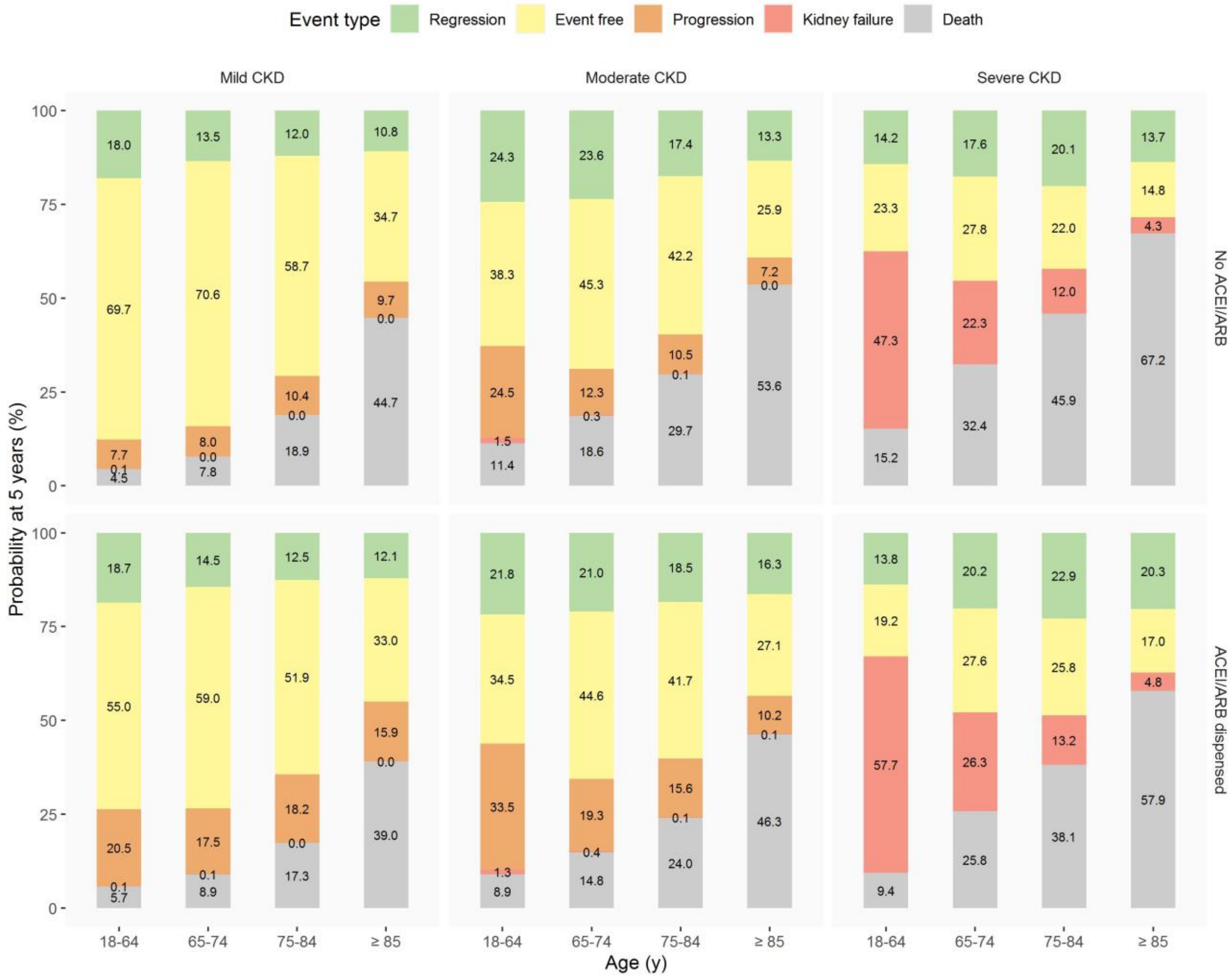

Abbreviations: ACEI/ARBs, angiotensin-converting enzyme inhibitor or angiotensin receptor blockers.

© 2021 Liu P et al. *JAMA Network Open*.

eFigure 12. Probabilities of Outcomes at 5 Years From Initial CKD Stage, by Diabetes, Cardiovascular Disease, and Age Categories at Baseline

A) Mild CKD

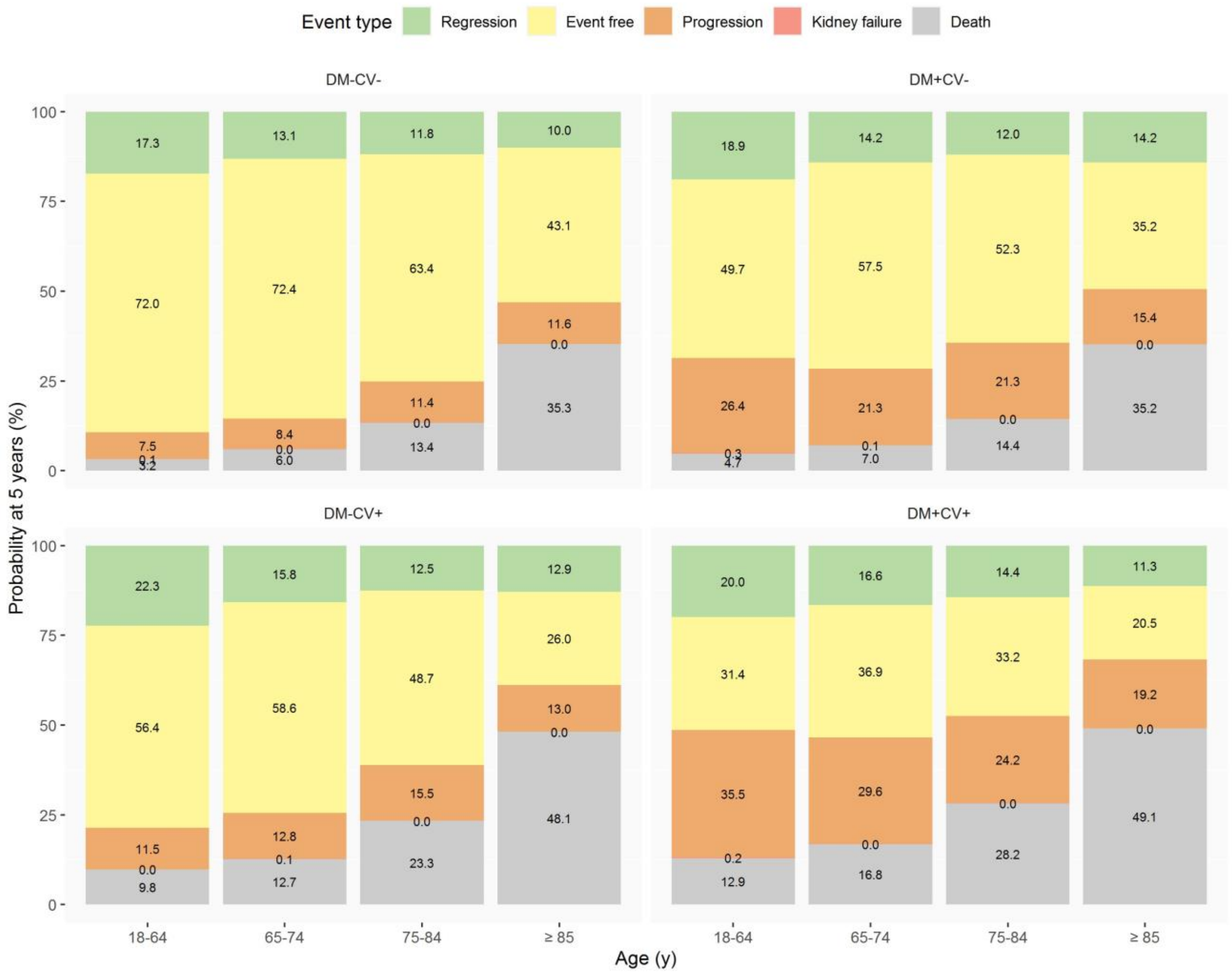

© 2021 Liu P et al. *JAMA Network Open*.

B) Moderate CKD

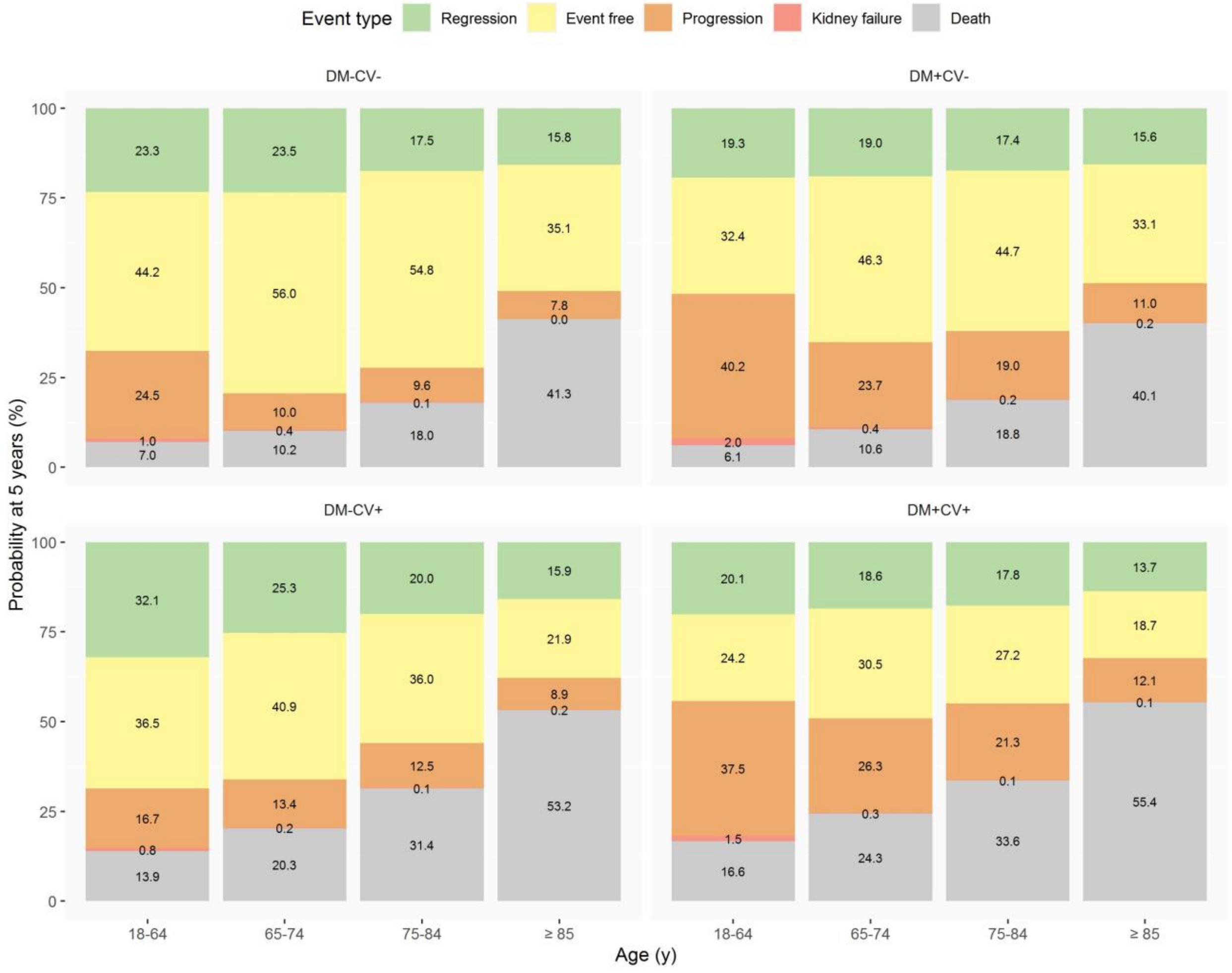

© 2021 Liu P et al. *JAMA Network Open*.

C) Severe CKD

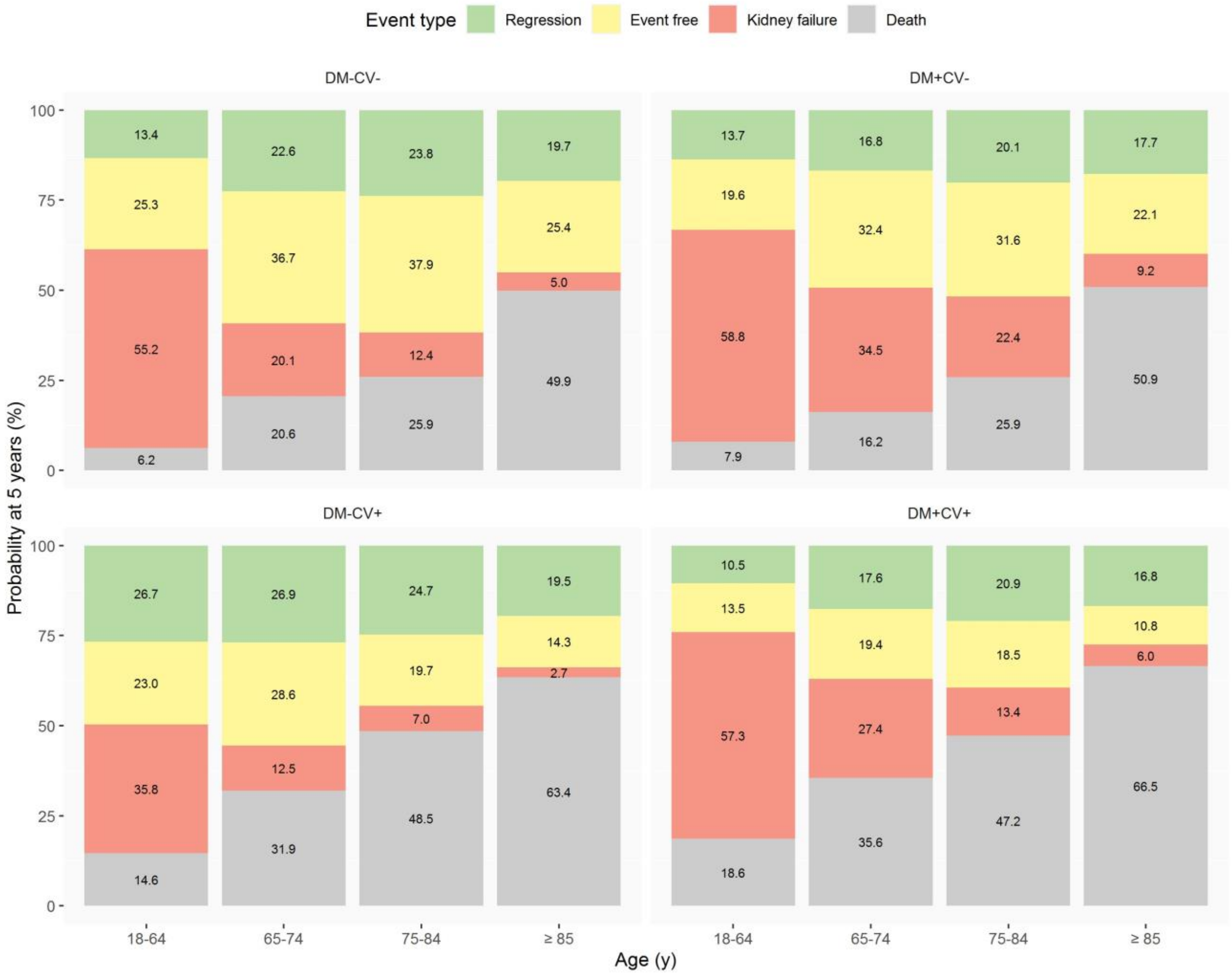

© 2021 Liu P et al. *JAMA Network Open*.

Abbreviations: DM-CV-, without diabetes and cardiovascular disease; DM+CV-, with diabetes and without cardiovascular disease; DM-CV+, without diabetes and with cardiovascular disease; DM+CV+, with diabetes and cardiovascular disease.

© 2021 Liu P et al. *JAMA Network Open*.

eFigure 13. Cumulative Incidence Functions Over Time Following Regression

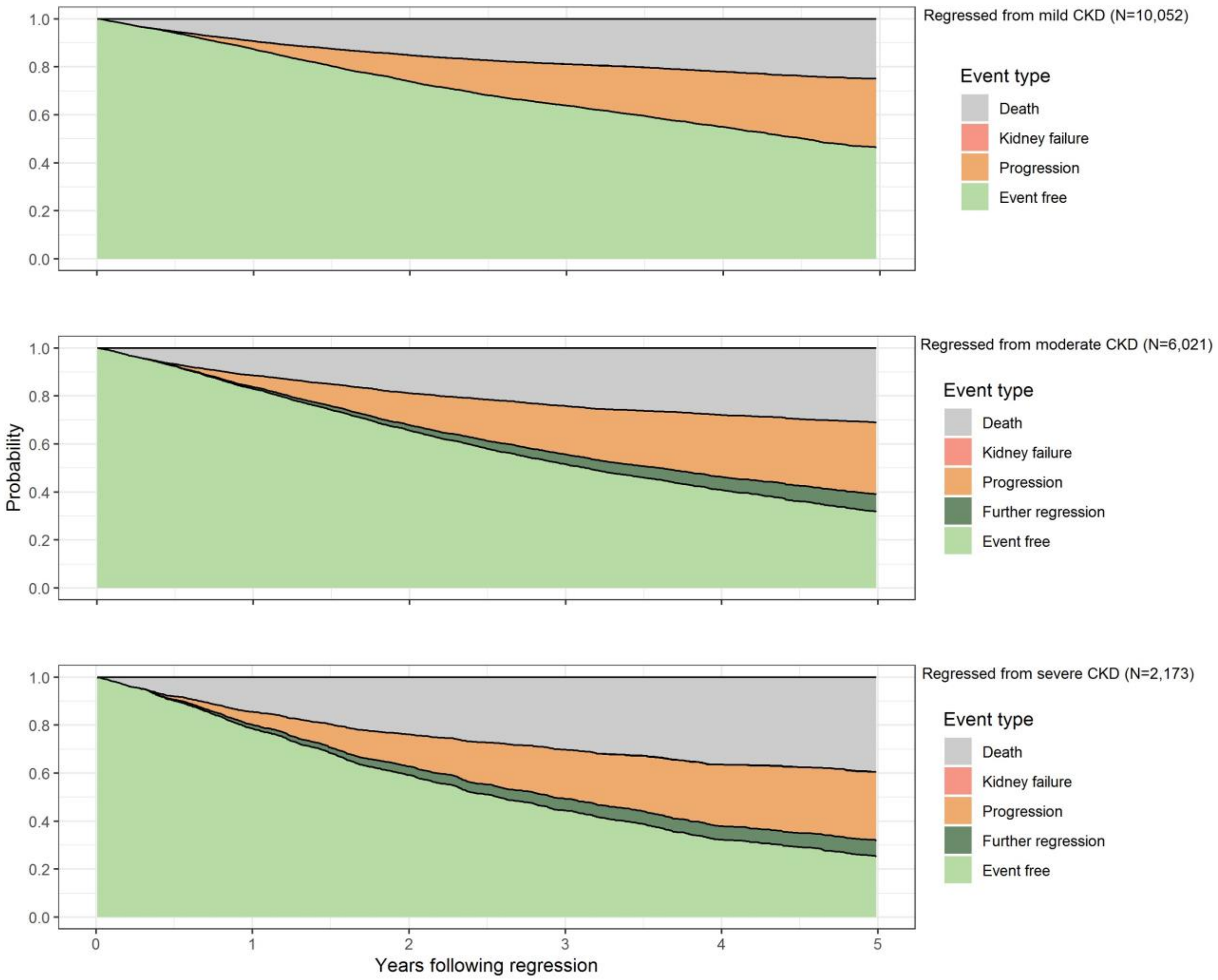

© 2021 Liu P et al. *JAMA Network Open*.

eFigure 14. Probabilities of Outcomes at 5 Years Following Regression

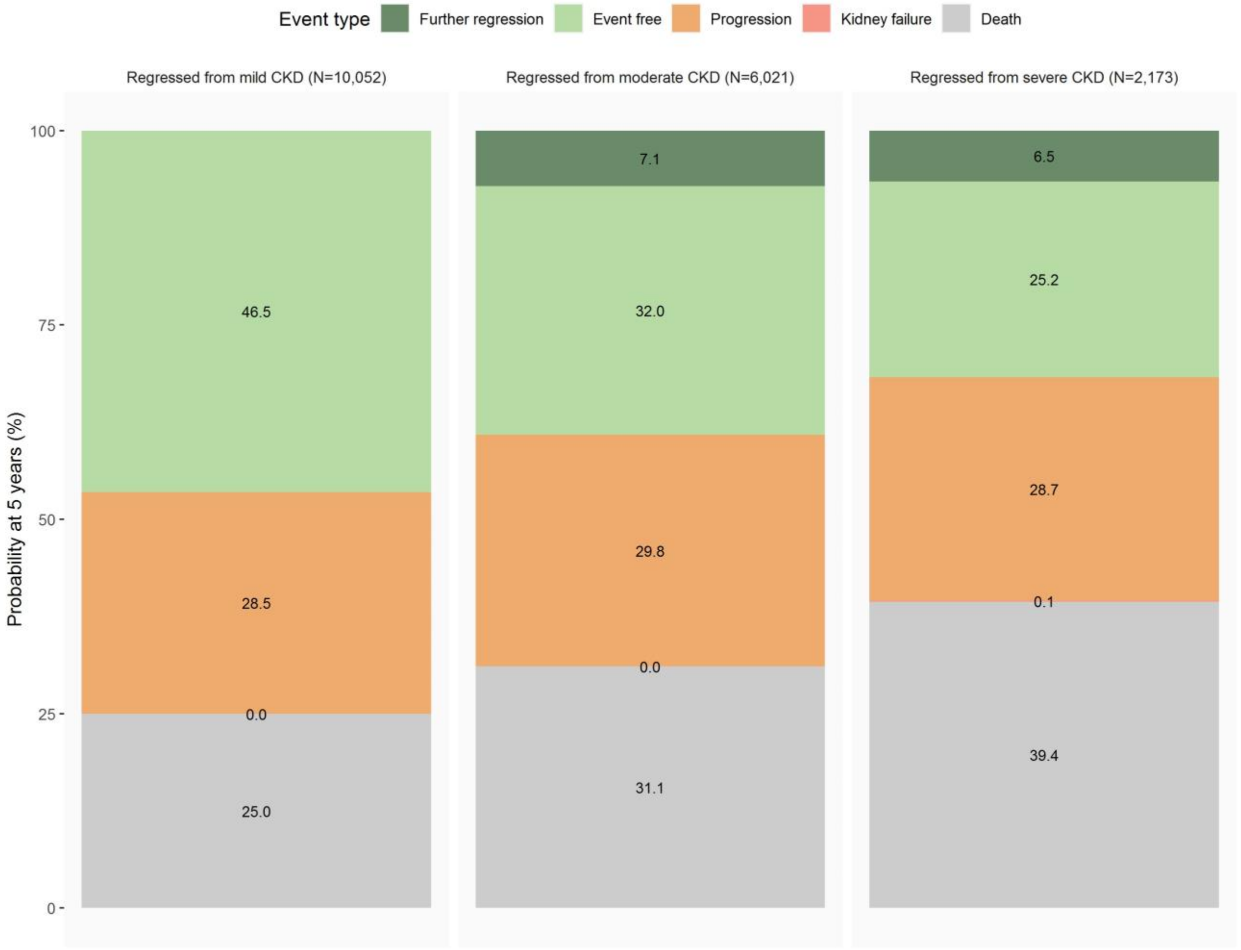

© 2021 Liu P et al. *JAMA Network Open*.

eFigure 15. Probabilities of Outcomes at 5 Years Following Regression, by Age Categories

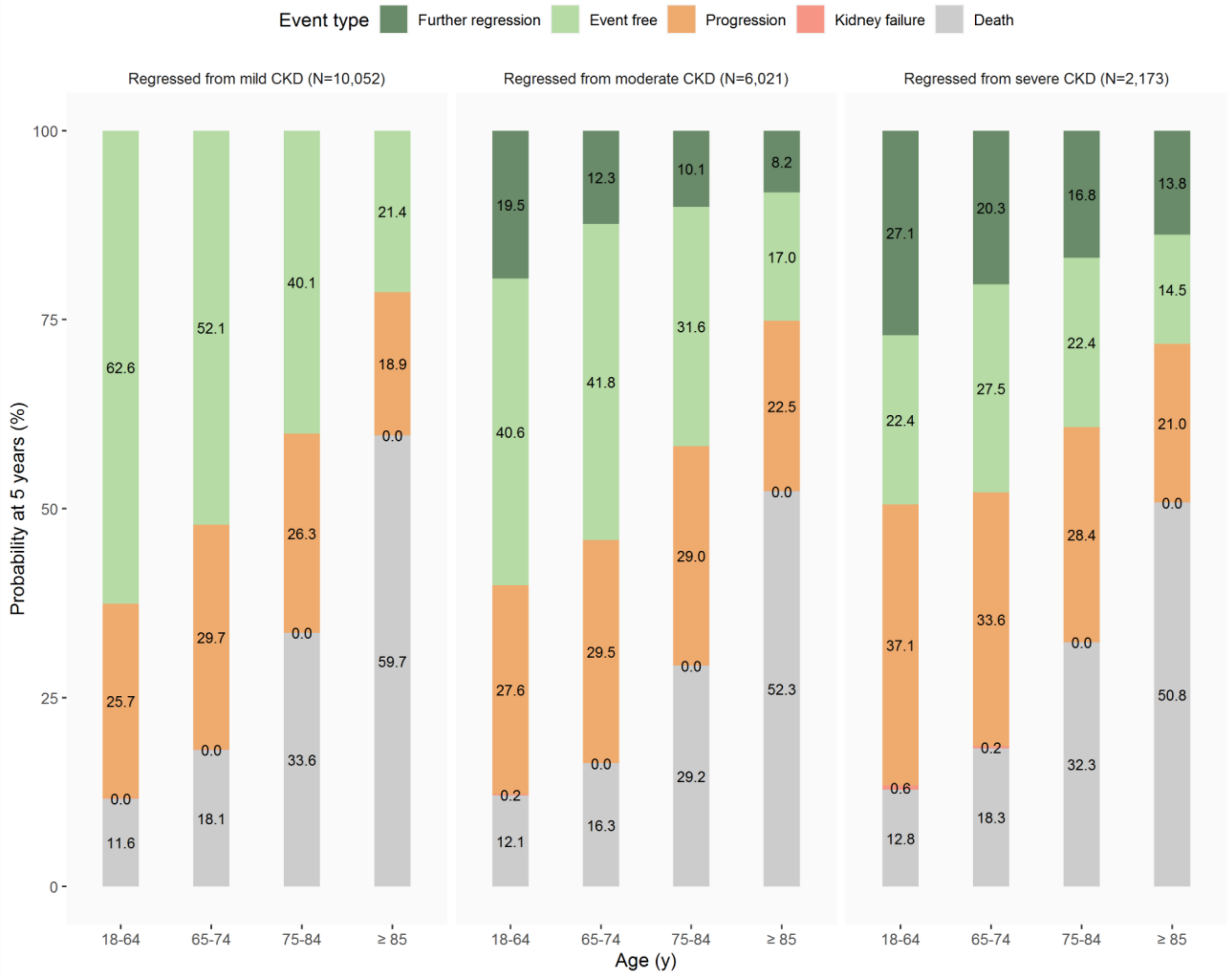

© 2021 Liu P et al. *JAMA Network Open*.

eFigure 16. Incidence of CKD, by CKD Stage and Age Categories (Moving Average eGFR Method)

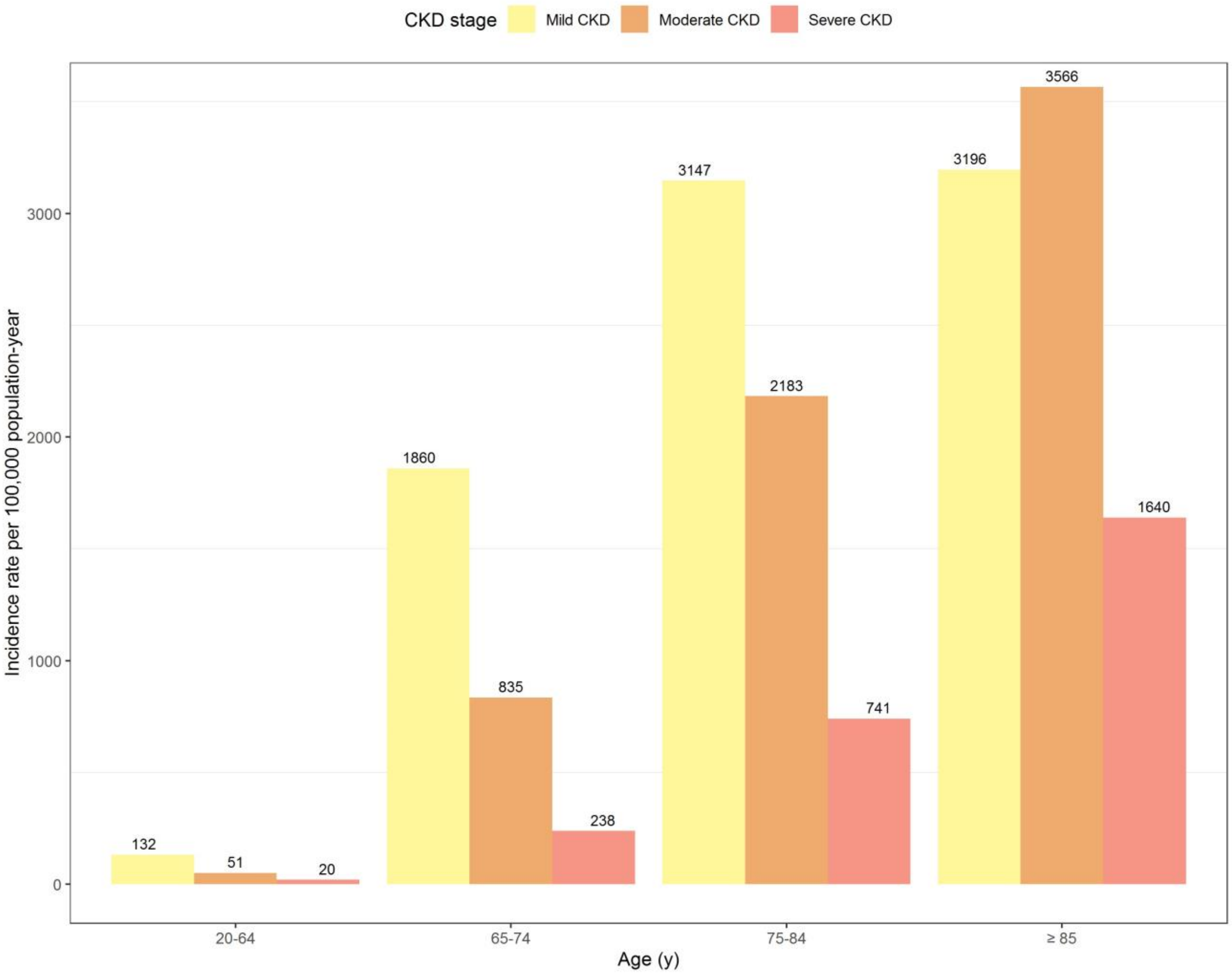

© 2021 Liu P et al. *JAMA Network Open*.

eFigure 17. Probabilities of Outcomes at 5 Years From Initial CKD Stage (Moving Average eGFR Method)

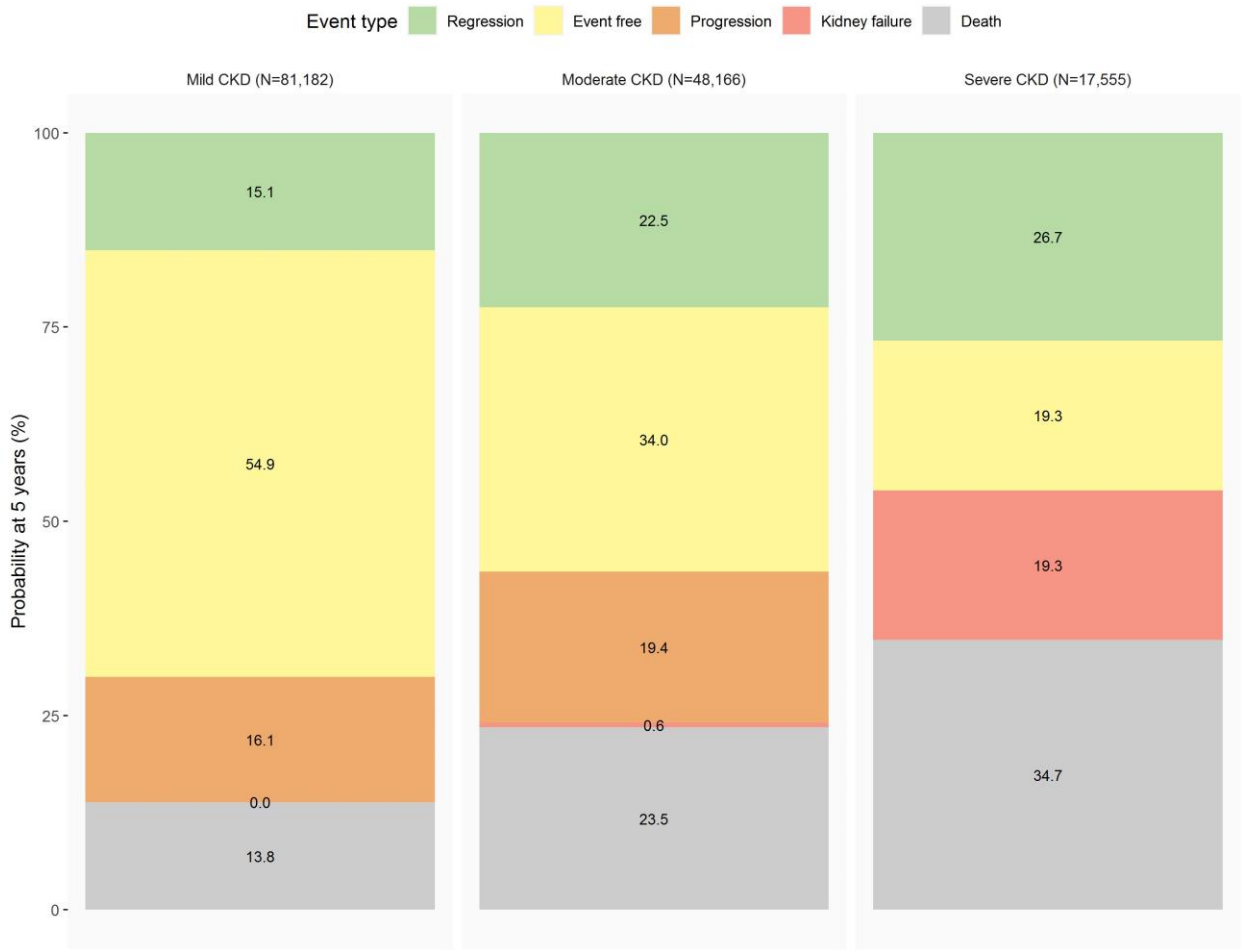

© 2021 Liu P et al. *JAMA Network Open*.

eFigure 18. Probabilities of Outcomes at 5 Years From Initial CKD Stage, by Age Categories (Moving Average eGFR Method)

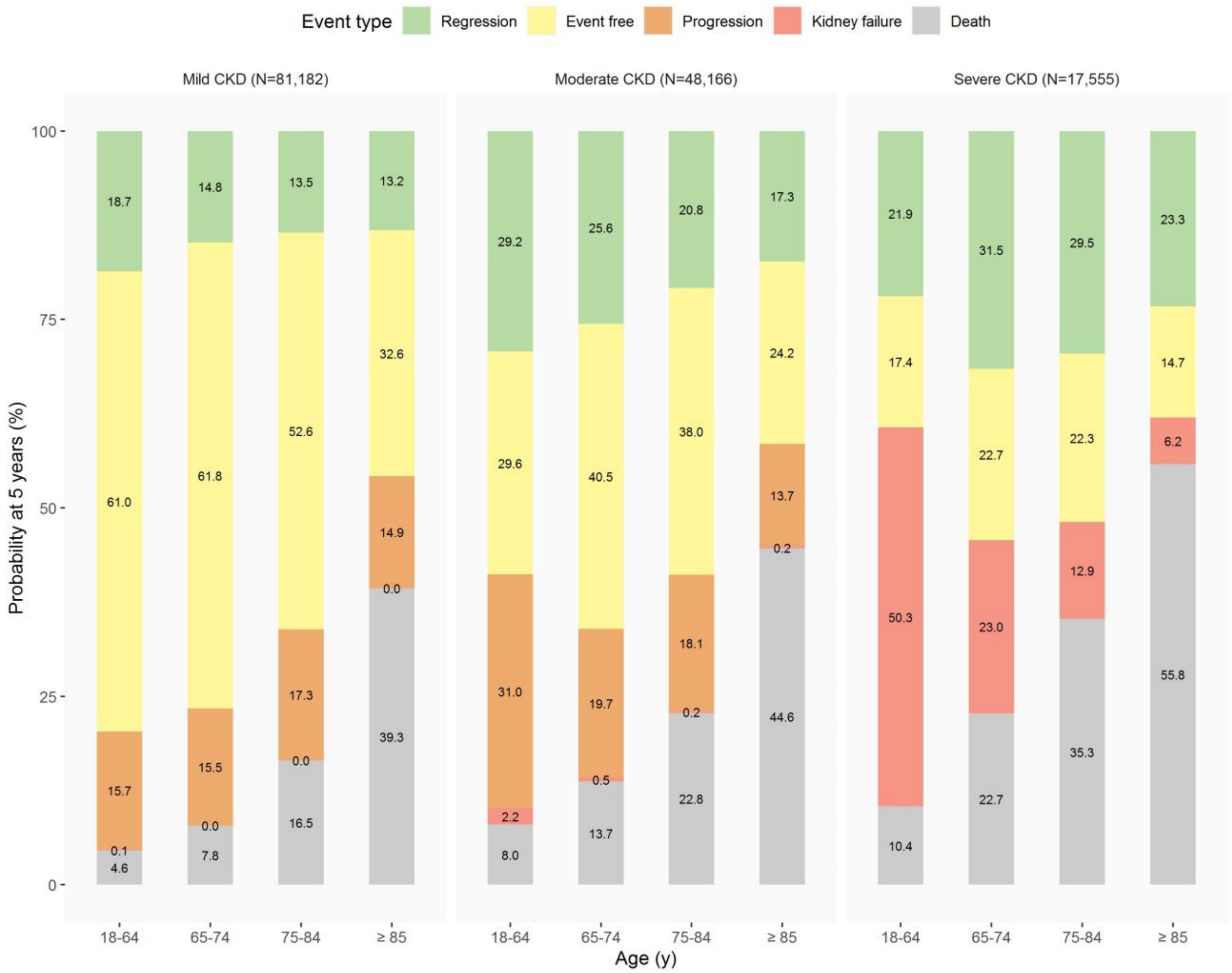

© 2021 Liu P et al. *JAMA Network Open*.

eFigure 19. Probabilities of Outcomes at 5 Years From Initial CKD Stage (91-455 Days Chronicity Criterion)

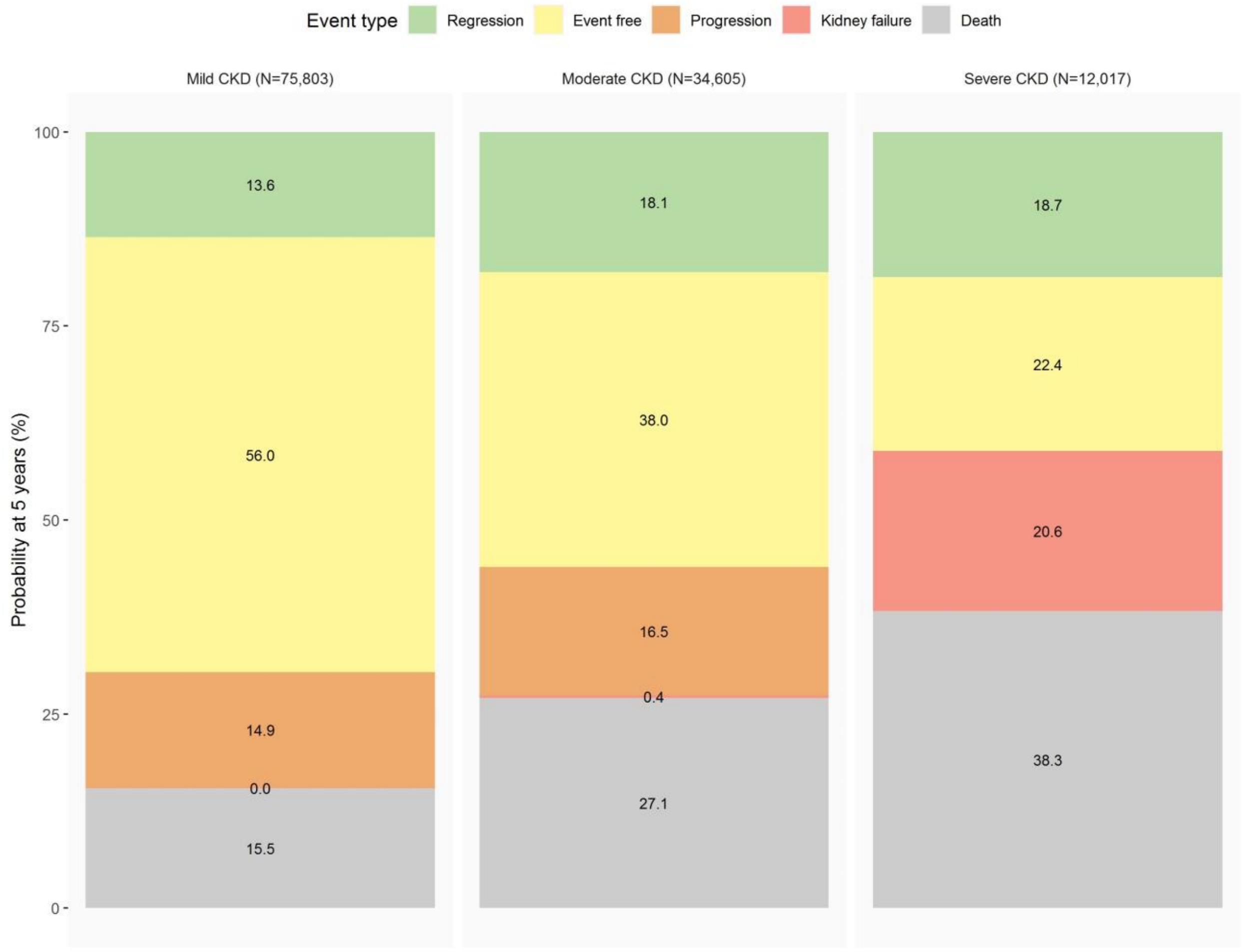

© 2021 Liu P et al. *JAMA Network Open*.

eFigure 20. Probabilities of Outcomes at 5 Years From Initial CKD Stage, by Age Categories (91-455 Days Chronicity Criterion)

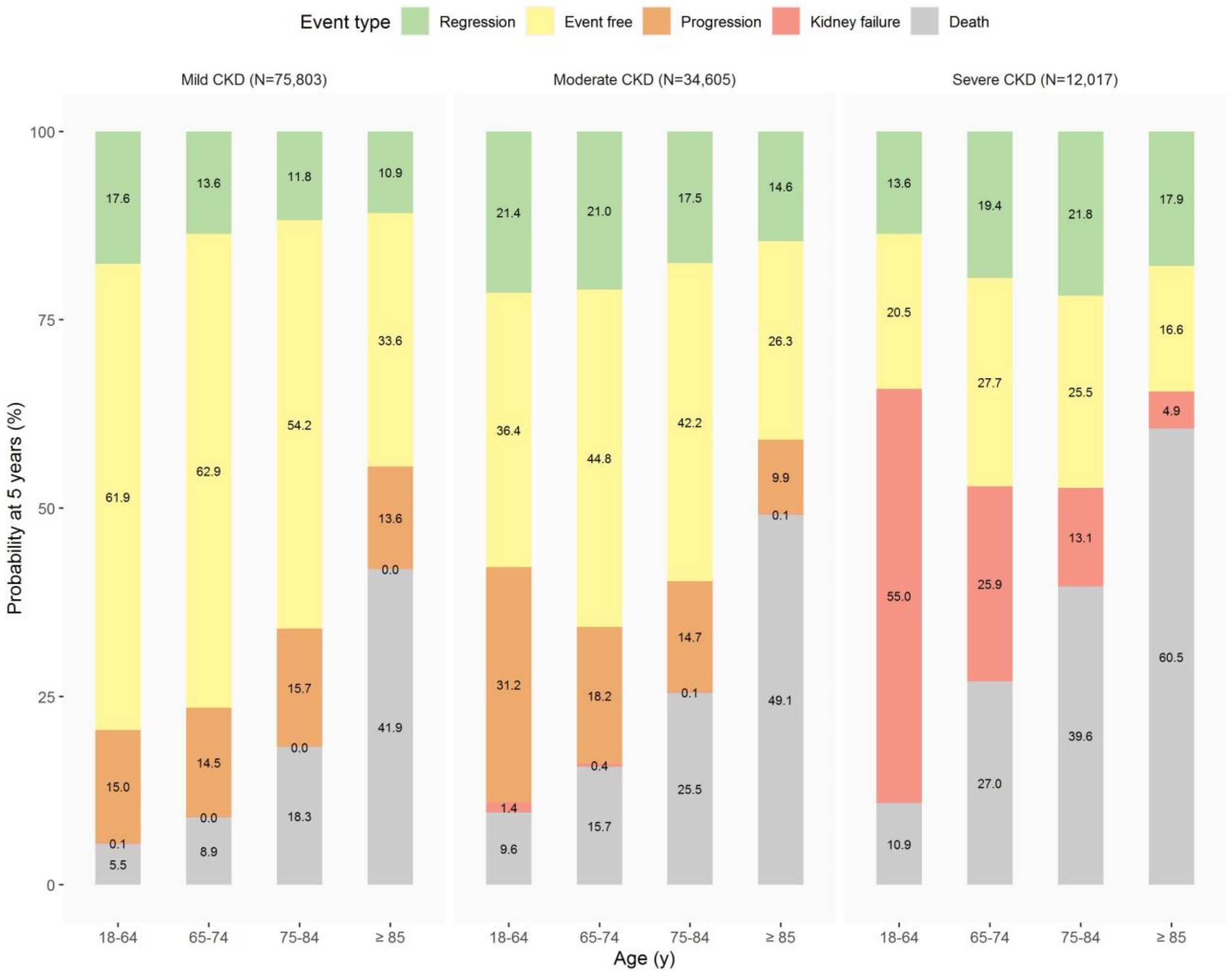

© 2021 Liu P et al. *JAMA Network Open*.

eReferences

1. Kidney Disease: Improving Global Outcomes (KDIGO) CKD Work Group. KDIGO 2012 Clinical Practice Guideline for the Evaluation and Management of Chronic Kidney Disease. *Kidney Int Suppl.* 2013;3(1):112-119.
2. Tonelli M, Vachharajani TJ, Wiebe N, et al. Methods for identifying 30 chronic conditions: application to administrative data. *BMC Med Inform Decis Mak.* 2015;15:31.

© 2021 Liu P et al. *JAMA Network Open*.
